# Precision Screening for MetS: The Role of Derived Lipid Indicators in Chinese Populations

**DOI:** 10.1155/ije/9990629

**Published:** 2025-10-29

**Authors:** Jiayu Zhou, Weifang Dai, Weina Xu, Shanna Liu, Qingli Zhou

**Affiliations:** ^1^School of Medicine, Shihezi University, Shihezi 832000, Xinjiang, China; ^2^Department of Information Technology, The Fourth Affiliated Hospital of School of Medicine, and International School of Medicine, International Institutes of Medicine, Zhejiang University, Yiwu 322000, China; ^3^Department of Geriatric Geriatrics, Center for Regeneration and Aging Medicine, The Fourth Affiliated Hospital of School of Medicine, and International School of Medicine, International Institutes of Medicine, Zhejiang University, Yiwu 322000, China

**Keywords:** blood lipids, CHARLS, metabolic syndrome, middle-aged and elderly people, obesity

## Abstract

Metabolic syndrome (MetS) is a significant public health concern among middle-aged and elderly populations in China. This study evaluates the predictive value of four derived lipid indicators—lipid accumulation product (LAP), visceral adiposity index (VAI), triglyceride–glucose (TyG) index, and Chinese visceral adiposity index (CVAI) for MetS in Chinese adults aged ≥ 45 years. Data were sourced from the 2015 China Health and Retirement Longitudinal Study (CHARLS). Participants were classified according to the International Diabetes Federation (IDF), National Cholesterol Education Program (NCEP ATP III, 2001), and China's 2020 Guidelines for Type 2 Diabetes Prevention and Treatment. Multivariate logistic regression and ROC curve analyses assessed the predictive performance of these indicators. Results demonstrated that LAP and CVAI showed the highest predictive accuracy for MetS under the IDF criteria (AUC = 0.903), while LAP and TyG were most effective under the NCEP ATP III and China 2020 guidelines (AUC = 0.860). Subgroup analyses revealed sex- and age-specific variations in indicator effectiveness. This study suggests that derived indicators, particularly LAP and TyG, enhance the screening and management of MetS in middle-aged and elderly Chinese populations. These findings support the adoption of LAP and TyG in clinical practice to improve early detection and targeted intervention strategies.

## 1. Background and Aims

Metabolic syndrome (MetS) is a cluster of interrelated metabolic disorders, including central obesity, hypertension, elevated fasting blood glucose (FBG), hypertriglyceridemia, and low high-density lipoprotein cholesterol (HDL-C). Together, these conditions significantly elevate the risk of cardiovascular diseases, Type 2 diabetes, stroke, and overall mortality [1–8]. Affecting approximately 25% of adults globally and around 33.38% of the Chinese population, the prevalence of MetS continues to grow, particularly among middle-aged and elderly adults in China [9, 10]. This trend highlights the urgent need for targeted public health interventions and policy initiatives to mitigate its impact.

While established diagnostic criteria for MetS, including those from the World Health Organization (WHO), International Diabetes Federation (IDF), and National Cholesterol Education Program Adult Treatment Panel III (NCEP ATP III), are extensively utilized, their implementation in large-scale screenings is frequently constrained by the intricate clinical evaluations they necessitate and their inconsistent applicability across diverse population [11, 12]. Moreover, gold-standard methods for assessing body fat distribution, such as computed tomography (CT) or magnetic resonance imaging (MRI), are costly and impractical for widespread use, highlighting the need for more accessible, cost-effective screening methods.

Derived indicators such as the lipid accumulation product (LAP), visceral adiposity index (VAI), triglyceride-glucose (TyG) index, and Chinese visceral adiposity index (CVAI) may offer a more nuanced understanding of MetS risk by integrating multiple metabolic parameters into a single score. By combining several components of MetS, these indicators potentially enhance predictive accuracy and clinical utility over individual components. They provide an integrated risk assessment that captures the combined effects of multiple metabolic factors and reflects underlying pathophysiological mechanisms, such as abdominal fat distribution and insulin resistance. This approach might overcome the limitations of traditional measures such as body mass index (BMI) and waist circumference (WC), which can vary significantly with age, sex, and ethnicity and often fail to distinguish between fat and muscle mass [13–23].

This study aims to evaluate and compare the effectiveness of these derived indicators—LAP, VAI, TyG, CVAI, BMI, and WC—in predicting MetS among middle-aged and elderly Chinese adults. By identifying the most accurate predictive cutoffs across various diagnostic criteria, this study seeks to improve early detection, intervention, and personalized treatment strategies, enhancing MetS screening practices and contributing to its prevention and management in China.

## 2. Materials and Methods

### 2.1. Data Sources

This study utilized data from the 2015 wave of the China Health and Retirement Longitudinal Study (CHARLS), a nationally representative longitudinal survey targeting individuals aged 45 years and older across 28 provinces in China. The CHARLS project, initiated in 2011 and conducted biennially, collects extensive social, economic, and health information, with the 2015 wave (Wave 3) being particularly valuable due to its inclusion of detailed blood test data. This wave was selected because it provides comprehensive clinical and lipid-related biomarkers essential for investigating MetS and its indicators, which were not consistently available in other waves. Although more recent data (e.g., 2018 or 2020 waves) exist, the 2015 dataset remains the most appropriate for this study due to its unique combination of demographic, anthropometric, and laboratory measurements tailored to middle-aged and elderly populations at higher risk for MetS.

**Figure 1:**
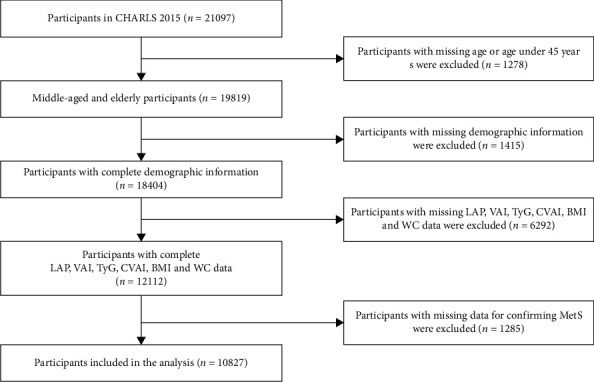
Flowchart depicting the inclusion and exclusion criteria in the CHARLS dataset.

Data collection involved face-to-face computer-assisted personal interviews (CAPI), structured questionnaires, physical measurements, and venous blood draws. The survey employed a multistage sampling strategy covering 150 county-level units and 450 village-level units to ensure representativeness. Participants under 45 were excluded to focus on the demographic most vulnerable to MetS, where prevalence and associated risks are significantly elevated. Ethical approval was obtained from the Institutional Review Board at Peking University (IRB00001052-11015), and all participants provided informed consent. Participant selection followed a systematic process to ensure data quality and minimize confounding, as detailed in Figure 1. The inclusion criteria required participants to meet all of the following conditions:

1. Aged ≥ 45 years: Aligning with CHARLS' focus on middle-aged and older adults at elevated MetS risk.
2. Complete anthropometric data: Precise measurements (height ±0.1 cm, weight ±0.1 kg, WC ±0.1 cm) for calculating BMI, WC, and derived indices (LAP, VAI, and CVAI).
3. Complete laboratory biomarkers: Fasting glucose and lipid profiles (triglycerides [TG] and HDL-C) for MetS definition and index calculation (TyG and CVAI).

Exclusion was applied sequentially:

  Step 1: Missing age information or age < 45 years (*n* = 1278)
  Step 2: Incomplete demographic data (sex/education/residence/economic status; *n* = 1415)
  Step 3: Missing derived indicator data (LAP/VAI/TyG/CVAI/BMI/WC; *n* = 12,112)
  Step 4: Incomplete MetS confirmation data (blood pressure/glucose/lipids; *n* = 1285)

### 2.2. Definition of MetS

MetS was defined according to three different sets of criteria: the NCEP ATP III (2001), IDF (2005), and China's 2020 Guidelines for the Prevention and Treatment of Type 2 Diabetes. According to the NCEP ATP III (2001) criteria, MetS is diagnosed when three or more of the following conditions are present: abdominal obesity (WC ≥ 102 cm for men and ≥ 88 cm for women), hyperglycemia (FBG ≥ 5.6 mmol/L or currently receiving treatment for diabetes), high TG (TG ≥ 150 mg/dL or on lipid-lowering therapy), low HDL-C (< 40 mg/dL for men and < 50 mg/dL for women or on treatment), and elevated blood pressure (≥ 130/85 mmHg or currently on antihypertensive treatment). In contrast, the IDF (2005) criteria require the presence of central obesity (WC ≥ 90 cm for men and ≥ 80 cm for women) as a mandatory condition, along with any two of the following: high TG (≥ 150 mg/dL or on treatment), low HDL-C (< 40 mg/dL for men, < 50 mg/dL for women, or on treatment), elevated blood pressure (≥ 130/85 mmHg or on treatment), or hyperglycemia (FBG ≥ 5.6 mmol/L or on treatment). The China (2020 Guidelines) use a similar approach but with modified cutoffs: MetS is diagnosed when three or more of the following are met: abdominal obesity (WC ≥ 90 cm for men and ≥ 85 cm for women), hyperglycemia (FBG ≥ 6.1 mmol/L or 2-h postglucose load ≥ 7.8 mmol/L or on treatment), high TG (≥ 150 mg/dL or on treatment), low HDL-C (< 40 mg/dL for men and < 50 mg/dL for women or on treatment), and elevated blood pressure (≥ 130/85 mmHg or on treatment).

These criteria differ primarily in their definitions of abdominal obesity, the required number of conditions for diagnosis, and specific cutoffs for blood glucose and lipids. The NCEP ATP III standard allows more flexibility in defining MetS by not mandating central obesity, whereas the IDF emphasizes central obesity as essential for diagnosis. The China 2020 Guidelines adjust these criteria further to better reflect the metabolic risk profiles specific to the Chinese population. Understanding these differences is crucial for this study, as they may affect the identification and classification of MetS in middle-aged and elderly Chinese adults. This study aims to evaluate the predictive values of various obesity and lipid-related indices (LAP, VAI, TyG, CVAI, BMI, and WC) using these different diagnostic criteria to identify the most effective cutoffs for MetS screening and prevention in this population.

### 2.3. Physiological Index Measurements

Anthropometric and clinical measurements were performed by trained researchers following standardized protocols. Weight and height were measured with participants wearing light clothing and no shoes; WC was measured at the navel level after exhalation. Blood pressure was measured three times at one-minute intervals using an automated electronic device, with the average value used for analysis. Blood samples were collected by the Chinese CDC, stored at −20°C, and analyzed for TG, fasting glucose (FBG), and HDL-C using enzyme colorimetric assays. Participants fasted for at least 8 h before blood collection. The formula [21, 24–27] was as follows:

(1) $$\begin{array}{c} \text{BMI}=\frac{\text{Weight }\left(\text{kg}\right)}{\text{Height }{\left(m\right)}^{2}}, \end{array}$$

(2) $$\begin{array}{c} \text{LAP}\left(\text{male}\right)=\left[\left(\text{WC }\left(\text{cm}\right)-65\right)\times \text{TG }\left(\text{mmol}/L\right)\right], \end{array}$$

(3) $$\begin{array}{c} \text{VAI }\left(\text{male}\right)=\frac{\text{WC }\left(\text{cm}\right)}{\left(39.68+1.88\times \text{BMI}\left(\text{kg}/m^{2}\right)\right)}\times \frac{\text{TG }\left(\text{mmol}/L\right)}{1.03}\times \frac{\;1.31}{\text{HDL}-C\left(\text{mmol}/L\right)},\\ \text{VAI }\left(\text{female}\right)=\frac{\text{WC }\left(\text{cm}\right)}{\left(36.58+1.89\times \text{BMI}\left(\text{kg}/m^{2}\right)\right)}\times \frac{\text{TG }\left(\text{mmol}/L\right)}{0.81}\times \frac{1.52}{\text{HDL}-C\left(\text{mmol}/L\right)}, \end{array}$$

(4) $$\begin{array}{c} \text{CVAI }\left(\text{male}\right)=-267.93+0.68\times \text{age}+0.03\times \text{BMI }\left(\text{kg}/m^{2}\right)+4.00\times \text{WC }\left(\text{cm}\right)+22.00\times \log_{10}\text{TG }\left(\text{mmol}/L\right)-16.32\times \text{HDL}-C\;\left(\text{mmol}/L\right),\\ \text{CVAI }\left(\text{female}\right)=-187.32+1.71\times \text{age}+4.23\times \text{BMI }\left(\text{kg}/m^{2}\right)+1.12\times \text{WC }\left(\text{cm}\right)+39.76\times \log_{10}\text{TG }\left(\text{mmol}/L\right)-11.66\times \text{HDL}-C\;\left(\text{mmol}/L\right), \end{array}$$

(5) $$\begin{array}{c} \text{TyG index}=\text{Ln }\left[\frac{\text{TG }\left(\text{mg}/\text{dL}\right)\times \text{FBG }\left(\text{mg}/\text{dL}\right)}{2}\right]. \end{array}$$

### 2.4. Statistical Analysis

Data were analyzed using appropriate statistical methods based on their distribution. Normally distributed data are expressed as the mean ± standard deviation (SD) and were compared between two groups using an independent *t*-test. Non-normally distributed data are presented as the median (P25, P75) and were compared using the Mann–Whitney U test. Categorical data are expressed as frequencies (percentages) and were analyzed using chi-square tests. The strength of correlations was evaluated using Cramer's V coefficient, where values between 0.1 and 0.3 indicate weak correlation, 0.3 to 0.6 indicate moderate correlation, and values ≥ 0.6 indicate strong correlation.

Multivariate logistic regression was performed to calculate the odds ratios (ORs) and 95% confidence intervals (CI) for the risk of MetS across quartiles of six indices: LAP, VAI, CVAI, TyG, BMI, and WC. Four models were developed: Model 1 was unadjusted; Model 2 was adjusted for age, sex, marital status, education level, smoking, and drinking; Model 3 further adjusted for systolic blood pressure (SBP), diastolic blood pressure (DBP), TG, HDL-C, and FBG; and Model 4 additionally adjusted for place of residence, social activities, and health insurance. The selected variables showed no multicollinearity (variance inflation factor, VIF < 5). We employed a directed acyclic graph (DAG) approach to identify potential confounders, guided by established MetS pathophysiology literature. Our hierarchical modeling strategy was designed to systematically evaluate confounding at multiple levels (Figure S1).

The diagnostic performance of each logistic regression model was assessed using ROC curve analysis. The area under the ROC curve (area under the curve [AUC]), sensitivity, specificity, positive likelihood ratio (LR+), negative likelihood ratio (LR−), Youden index, and corresponding critical values were calculated to evaluate the predictive value of each indicator for MetS. Youden's index was used to determine the optimal cutoff for each indicator. An AUC > 0.9 indicated high diagnostic accuracy, 0.7 to 0.9 indicated moderate accuracy, and 0.5 to 0.7 indicated low accuracy. AUC values were compared using the DeLong test.

Subgroup analyses were conducted to compare the predictive performance of the six indicators (LAP, VAI, CVAI, TyG, WC, and BMI) across different populations stratified by sex and age.

Data were analyzed using Stata MP18 and MedCalc Version 19.0.

## 3. Results

### 3.1. Baseline Data Analysis

A total of 10,827 participants were included in the analysis, with 47.03% males and 52.97% females. Among the participants, 45.42% were aged below 60 years, and 54.58% were aged 60 years or older. The baseline characteristics of the study population, including sex, age, place of residence, smoking and drinking history, social activity participation, health insurance status, marital status, and education level, as well as biological data such as TG, HDL-C, FBG, WC, BMI, SBP, DBP, LAP, VAI, CVAI, and TyG, are presented in Table 1. There were significant differences between participants with and without MetS in terms of age, sex, smoking status, drinking status, marital status, and education level (*p* < 0.05). Male participants showed a higher prevalence of current smoking and alcohol consumption compared to females (*p* < 0.001). Participants aged ≥ 60 years were more likely to be married, have lower educational levels, and have higher SBP, DBP, and TG levels (*p* < 0.05). Additionally, significant differences were observed in LAP, VAI, CVAI, TyG, WC, and BMI between different age and sex groups (*p* < 0.001).

### 3.2. Correlations of Obesity and Lipid-Related Indicators With MetS and Its Components

We analyzed the distribution of obesity and lipid-related indicators across different groups to assess their correlations with MetS and its components. The relationships between each indicator and MetS, as well as its components, were evaluated according to different diagnostic criteria due to varying definitions of hyperglycemia and low HDL-C (Table 2). Cramer's V coefficient was used to determine the strength of these associations (Table 3). Under the IDF criteria, MetS showed a strong association with LAP, CVAI, and WC. Abdominal obesity was also strongly correlated with LAP, CVAI, BMI, and WC. According to the China 2020 criteria, MetS was most strongly associated with LAP, while abdominal obesity was again strongly associated with LAP, CVAI, BMI, and WC. Under the NCEP ATP III criteria, abdominal obesity was significantly associated with CVAI. Across all criteria, TG abnormalities demonstrated a strong correlation with LAP, VAI, and TyG.

Table 2 presents the prevalence of MetS and its components across quartiles of the six indicators (LAP, VAI, CVAI, TyG, BMI, and WC). Notably, higher quartiles of LAP, VAI, CVAI, TyG, and BMI were associated with a greater prevalence of MetS across all three diagnostic standards, with significant differences between the lowest (Q1) and highest (Q4) quartiles (*p* < 0.001). Table 3 shows the Cramer's V coefficients indicating the strength of the correlations between the indicators and MetS components. The strongest correlations were observed between elevated TG and LAP, VAI, and TyG (Cramer's V > 0.7), regardless of the diagnostic criteria used. Elevated WC showed strong correlations with LAP, CVAI, BMI, and WC across all criteria (Cramer's V > 0.6).

### 3.3. Multivariate Logistic Regression Analysis of the Effect of Obesity and Lipid-Related Indicators on MetS Risk in Middle-Aged and Elderly People

Table 4 shows the associations between various obesity-related indicators and the risk of MetS in middle-aged and elderly adults. The data were divided into quartiles for each indicator, and the ORs indicated an increased likelihood of developing MetS with each rise in quartile. Under the IDF, the unadjusted model (Model 1) showed a 6.144-fold increase in MetS risk for each quartile increase in LAP (*p* < 0.001), while adjusted models showed OR of 6.080, 6.925, and 6.747 for Models 2, 3, and 4, respectively, after accounting for demographic, clinical, and socioeconomic factors. Overall, LAP, VAI, CVAI, and TyG demonstrated strong predictive value for MetS, with AUC values ranging from 0.796 to 0.933, indicating moderate to high accuracy.

### 3.4. ROC Curves of Obesity and Lipid-Related Indicators for Predicting MetS Risk

**Figure 2:**
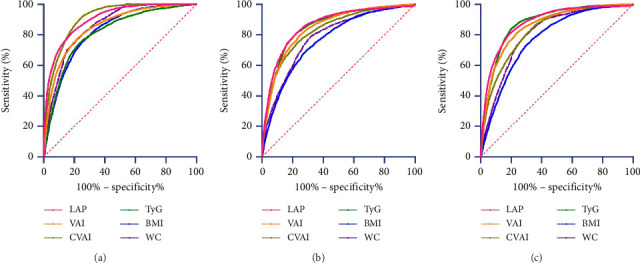
ROC curve of body fat index to predict the risk of MetS. Note: (a) under the IDF standard; (b) under the NCEP ATP III standard; (c) under the prevention and treatment of Type 2 diabetes in China (2020) standard.

ROC curves were used to evaluate the predictive ability of LAP, VAI, CVAI, TyG, BMI, and WC for MetS (Table 5, Figure 2). LAP, VAI, CVAI, and TyG demonstrated high accuracy in predicting MetS across three diagnostic criteria, with AUC values greater than 0.800. The AUCs were 0.903, 0.854, 0.909, and 0.815 under the IDF criteria; 0.862, 0.846, 0.832, and 0.856 under the NCEP ATP III criteria; and 0.860, 0.836, 0.823, and 0.853 according to the China 2020 Edition. WC had greater predictive power (AUC > 0.800) according to the IDF criteria, while the predictive powers of WC and BMI were significantly lower than those of LAP, VAI, CVAI, and TyG under the NCEP ATP III and China 2020 criteria. Under these standards, the AUC values for LAP were 0.903 (95% CI: 0.898–0.909), 0.862 (95% CI: 0.854–0.870), and 0.881 (95% CI: 0.874–0.888), respectively. Pairwise comparisons of the AUCs for the six body fat metrics in predicting MetS, analyzed using the DeLong test, are shown in Table 6.

### 3.5. Subgroup Analyses

**Figure 3:**
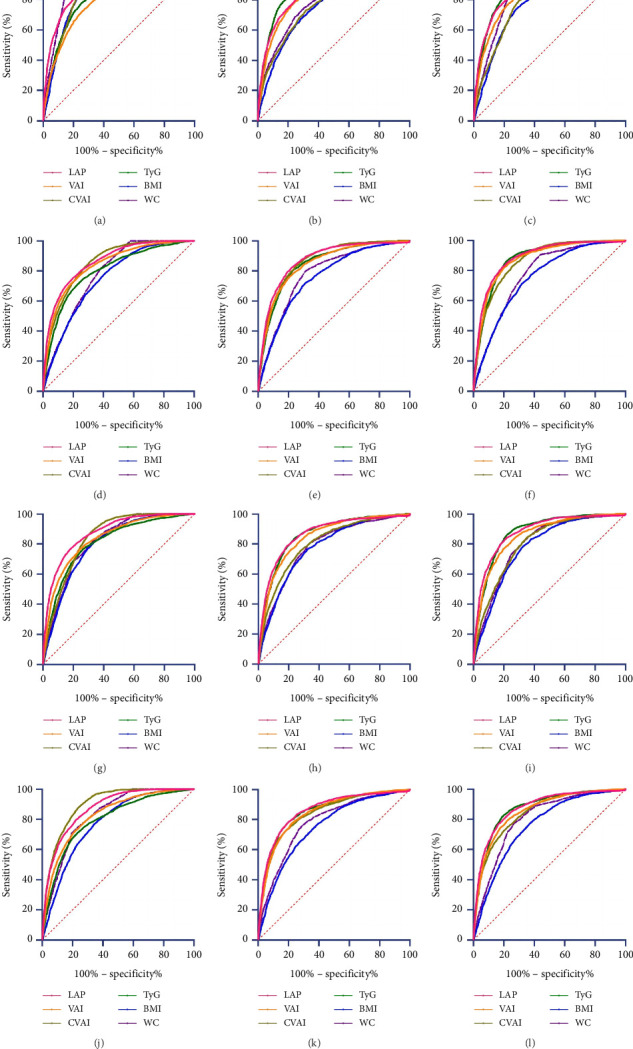
ROC curve of body fat index for predicting the risk of MetS. Note: (a–c) ROC curves for male subjects' body fat indices predicting MetS risk under (a) IDF, (b) NCEP ATP III, and (c) China (2020); (d–f) ROC curves for female subjects' body fat indices predicting MetS risk under (d) IDF, (e) NCEP ATP III, and (f) China (2020); (g–i) ROC curves for subjects aged < 60 predicting MetS risk under (g) IDF, (h) NCEP ATP III, and (i) China (2020); (j–l) ROC curves for subjects aged ≥ 60 predicting MetS risk under (j) IDF, (k) NCEP ATP III, and (l) China (2020). Abbreviations: AUC, area under the curve; BMI, body mass index; LAP, lipid accumulation product; CVAI, Chinese visceral adiposity index; MetS, metabolic syndrome; TyG, triglyceride glucose; VAI, visceral adiposity index.

Subgroup analyses by sex and age showed significant differences in the predictive power of obesity-related indicators for MetS risk (Tables S1–S12, Figure 3). For both men and women, LAP, VAI, CVAI, and TyG had high accuracy in predicting MetS across different diagnostic criteria. In men, CVAI and LAP had the highest predictive values, while LAP showed the highest predictive power in women. Age-based analyses revealed that LAP consistently had the highest predictive accuracy in both age groups (≥ 60 and < 60 years), followed by TyG and CVAI.

## 4. Discussion

This study compared four novel indicators—LAP, VAI, CVAI, and TyG index—across three different diagnostic criteria (IDF, NCEP ATP III, and China 2020 standards) to determine which indicators most effectively predict MetS in middle-aged and elderly Chinese adults. The results demonstrate that while all four measures are highly accurate in predicting MetS, their predictive power varies depending on the diagnostic criteria applied. According to the IDF criteria, LAP and CVAI were the most effective predictors, whereas LAP and TyG were superior according to the China 2020 and NCEP ATP III standards. Additionally, our study highlighted the importance of sex and age in influencing the predictive power of these indicators.

Our findings align with previous studies conducted in various populations, which have consistently shown that LAP and TyG are superior predictors of MetS compared to other indicators [28, 29]. However, our study uniquely extends this understanding by comparing these indicators across multiple diagnostic criteria, revealing that LAP consistently performs well regardless of the criteria used, while TyG demonstrates superior performance in specific subgroups, such as males and individuals over 60 years of age. This comprehensive comparison across different criteria provides new insights into the applicability of these indicators in different clinical and demographic settings.

MetS is closely linked with an increased risk of cardiovascular disease, stroke, and mortality, making the development of simple, effective predictors for clinical and screening evaluations critically important [25, 30]. Previous studies have established LAP and VAI as reliable predictive indicators across various populations, with particularly high predictive accuracy [31, 32]. Given the greater abdominal fat distribution observed in Asian populations, particularly among Chinese people, as compared to Western populations [33], it is crucial to examine indicators tailored to these specific characteristics. This study addressed this need by including CVAI and TyG, both of which were developed or adapted based on the unique physical and metabolic characteristics of the Chinese population.

The inclusion of CVAI and TyG in our analysis underscores their potential utility in predicting MetS risk in Chinese populations. CVAI, specifically designed for Chinese individuals, combines multiple metabolic parameters such as BMI, WC, serum TG, and HDL-C to provide a comprehensive measure of fat distribution relevant to MetS [34]. Similarly, the TyG index has been shown to outperform other indicators such as FBG, TG, atherogenic dyslipidemia, non-HDL cholesterol, and insulin resistance (HOMA-IR) in predicting MetS risk, further supporting its utility in clinical practice [35]. Given the sex-specific differences in MetS, where women generally store more adipose tissue, while men oxidize more lipids [36]. It is vital to consider sex-specific variations when evaluating MetS risk. The results from our sex-stratified analysis align with previous findings, such as those from a Mexican metabolomics study, which demonstrated significant sex-related differences in MetS-associated metabolites [37].

Our findings also highlight the importance of sex-specific differences in MetS risk, as men and women exhibit distinct metabolic patterns. For example, men typically have higher rates of lipid oxidation, while women tend to store more adipose tissue [38]. Our sex-stratified analyses are in line with other studies that highlight significant sex-related differences in MetS-associated factors. For instance, differences in fat distribution and metabolic profiles between men and women have been shown to influence MetS risk, underscoring the need for sex-specific evaluation strategies when selecting the most appropriate predictive indicators [39, 40]. These findings emphasize the need for sex-specific evaluation strategies when assessing MetS risk and selecting the most appropriate predictive indicators.

Our study also highlighted the impact of different diagnostic criteria on the prevalence and identification of MetS. By comparing the IDF, NCEP ATP III, and China 2020 standards, we observed significant variability in MetS prevalence. Under the IDF criteria, the prevalence was 40.55%, with women twice as likely as men to have MetS (43.50% vs. 27.40%). In contrast, the NCEP ATP III standard resulted in a lower overall prevalence of 28.30%, with 32.60% in women and 23.80% in men. The China 2020 standard showed a prevalence of 28.60%, with a slightly higher rate in women (29.92%) compared to men (27.03%). These differences underscore the importance of selecting appropriate diagnostic criteria based on the target population's specific characteristics, as these can significantly influence the identification and classification of MetS cases.

Given the observed differences in prevalence, we performed sex-based analyses to determine the most effective predictors for each group. Our findings indicate that the best predictors for MetS differ between men and women depending on the diagnostic criteria. Notably, LAP consistently demonstrated strong predictive efficacy across all groups and criteria (AUC > 0.850), aligning with previous studies conducted in various populations [41–43], age-stratified analysis (≥ 60 years vs. < 60 years) confirmed LAP as the strongest predictor of MetS regardless of age, likely due to its combined consideration of WC and lipid levels, factors known to vary with age and hormonal changes [44, 45]. Changes in fat distribution with age, especially in women postmenopause, further emphasize the importance of age-specific screening strategies [46–49].

Despite the valuable insights provided by this study, several limitations should be acknowledged. The reliance on self-reported data could introduce selection bias. Additionally, our sample consisted primarily of relatively healthy Chinese individuals, which may limit the generalizability of the findings to other ethnic groups. The study's cross-sectional design also precludes causal inferences. Future research should employ longitudinal approaches using diverse datasets to validate these findings and explore the longitudinal impact of these indicators on MetS risk in middle-aged and older adults.

## 5. Conclusions

In conclusion, our study confirms that LAP, VAI, CVAI, and TyG are significant predictors of MetS, with their effectiveness varying according to diagnostic criteria, sex, and age. Given the influence of sex and age on MetS incidence, tailored screening strategies incorporating these factors are recommended to improve early detection and management of MetS.

## Figures and Tables

**Table 1:** Baseline data analysis of subjects.

Variable	Total	Sex		*p* value	Age		*p* value
		Male *N* (%)	Female *N* (%)		< 60 *N* (%)	≥ 60 *N* (%)	
	(*n* = 10,827)	(*n* = 5092)	(*n* = 5735)		(*n* = 4918)	(*n* = 5909)	
Sex [*n* (%)]							
Male	5092 (47.03)				2139 (43.49)	2953 (49.98)	< 0.001^∗∗∗^
Female	5735 (52.97)				2779 (56.51)	2956 (50.03)	
Age (years)	60.90 ± 9.21	61.63 ± 9.22	60.24 ± 9.16	< 0.001^∗∗∗^			
Place of residence [*n* (%)]							
Urban	4060 (37.50)	1868 (36.68)	2192 (38.22)	0.099	1858 (37.78)	2202 (37.28)	0.582
Rural	6767 (62.50)	3224 (63.32)	3543 (61.78)		3060 (62.22)	3707 (62.73)	
Smoking history [*n* (%)]							
Never smoked	6087 (56.22)	855 (16.79)	5235 (91.28)	< 0.001^∗∗∗^	3003 (61.06)	3087 (52.24)	< 0.001^∗∗∗^
Quit smoking	1726 (15.94)	1515 (29.75)	211 (3.68)		582 (11.83)	1144 (19.36)	
Currently smoking	3014 (27.84)	2722 (53.46)	289 (5.04)		1333 (27.11)	1678 (28.40)	
History of alcohol consumption [*n* (%)]							
Never drank alcohol	5824 (53.79)	1378 (27.06)	4446 (77.52)	< 0.001^∗∗∗^	2710 (55.10)	3114 (52.70)	0.044^∗^
Drinking less than once a month	2143 (19.79)	1333 (26.18)	810 (14.12)		2196 (44.65)	2780 (47.05)	
More than once a month	2860 (26.42)	2381 (46.76)	479 (8.35)		12 (0.24)	15 (0.25)	
Participate in social activities [*n* (%)]							
No	5345 (49.37)	2472 (48.55)	2873 (50.10)	0.108	2200 (44.73)	3145 (53.22)	< 0.001^∗∗∗^
Yes	5482 (50.63)	2620 (51.45)	2862 (49.90)		2717 (55.24)	2764 (46.78)	
Participate in health insurance [*n* (%)]							
No	1452 (13.41)	659 (12.94)	800 (13.95)	0.125	363 (7.38)	560 (9.48)	< 0.001^∗∗∗^
Yes	9375 (86.59)	4433 (87.06)	4935 (86.05)		4555 (92.62)	5349 (90.52)	
Marital status [*n* (%)]							
Married, divorced, and widowed	10,759 (99.37)	5027 (98.72)	5732 (99.95)	< 0.001^∗∗∗^	4896 (99.55)	5863 (99.22)	0.03^∗^
Never married	68 (0.63)	65 (1.28)	3 (0.05)		22 (0.45)	46 (0.78)	
Educational level [*n* (%)]							
Illiterate	2777 (25.65)	579 (11.37)	2198 (38.33)	< 0.001^∗∗∗^	793 (16.12)	1984 (33.58)	< 0.001^∗∗∗^
Elementary school or below	4676 (43.19)	2443 (47.98)	2233 (38.94)		1954 (39.73)	2722 (46.07)	
Middle school or above	3374 (31.16)	2070 (40.65)	1304 (22.74)		2171 (44.14)	1203 (20.36)	
WC (cm)	85.73 ± 13.23	85.42 ± 13.36	86 ± 13.11	0.0238	86.60 ± 12.27	85.00 ± 13.94	< 0.001^∗∗∗^
[M (P25, P75)]	(79, 93.6)	(78.50, 96.60)	(79.80, 93.70)		(79.90, 94.00)	(78.50, 93.20)	
BMI (kg/m^2^)	24.56 ± 15.81	23.89 ± 12.11	36.20 ± 40.24	< 0.001^∗∗∗^	25.05 ± 12.47	24.15 ± 18.12	< 0.001^∗∗∗^
[M (P25, P75)]	(21.38, 26.35)	(20.86, 25.80)	(21.89, 26.78)		(21.99, 26.90)	(20.83, 25.82)	
SBP (mmHg)	131.50 ± 21.34	132.35 ± 21.02	130.74 ± 21.59	< 0.001^∗∗∗^	77.77 ± 12.62	76.14 ± 12.42	< 0.001^∗∗∗^
[M (P25, P75)]	(116, 145)	(117, 145)	(115, 144)		(69.00, 85.00)	(68.00, 83.00)	
DBP (mmHg)	76.88 ± 12.54	78.06 ± 12.92	75.84 ± 12.09	< 0.001^∗∗∗^	127.17 ± 19.55	135.11 ± 22.08	< 0.001^∗∗∗^
[M (P25, P75)]	(68, 84)	(69, 00, 86.00)	(68, 00, 83.00)		(113.00, 139.00)	(119.00, 149.00)	
TG (mg/dL)	145.02 ± 92.43	138.52 ± 93.72	150.79 ± 90.9	< 0.001^∗∗∗^	153.78 ± 99.25	137.72 ± 85.68	< 0.001^∗∗∗^
[M (P25, P75)]	(84.07, 172.57)	(77.88, 164.60)	(89.38, 179.65)		(86.73, 183.19)	(81.42, 163.72)	
HDL-C(mg/dL)	51.14 ± 11.60	49.97 ± 12.44	52.17 ± 10.69	< 0.001^∗∗∗^	50.78 ± 10.94	51.43 ± 12.12	0.0633
[M (P25, P75)]	(42.86, 57.53)	(41.31, 55.98)	(44.79, 58.30)		(43.24, 56.76)	(42.86, 57.92)	
FBG(mg/dL)	105.05 ± 36.38	104.95 ± 36.05	105.15 ± 36.68	0.7757	104.11 ± 35.83	105.83 ± 36.82	< 0.001^∗∗∗^
[M (P25, P75)]	(88.29, 108.11)	(88.29, 108.11)	(88.29, 108.11)		(88.29, 106.31)	(88.29, 108.11)	
LAP	43.74 ± 42.39	36.20 ± 40.24	50.44 ± 43.11	< 0.001^∗∗∗^	47.62 ± 44.51	40.52 ± 40.25	< 0.001^∗∗∗^
[M (P25, P75)]	(17.46, 58.25)	(12.78, 47.45)	(23.51, 66.72)		(19.36, 62.40)	(16.06, 54.84)	
VAI	5.21 ± 4.51	4.07 ± 4.00	6.21 ± 5.00	< 0.001^∗∗∗^	5.51 ± 4.59	4.95 ± 4.43	< 0.001^∗∗∗^
[M (P25, P75)]	(2.37, 6.39)	(1.85, 4.86)	(3.09, 7.59)		(2.58, 6.75)	(2.23, 6.08)	
CVAI	144.20 ± 78.50	116.54 ± 59.69	168.76 ± 84.80	< 0.001^∗∗∗^	140.18 ± 54.01	147.55 ± 94.03	< 0.001^∗∗∗^
[M (P25, P75)]	(111.78, 178.84)	(82.23, 154.98)	(140.73, 190.74)		(112.20, 170.83)	(111.25, 185.51)	
TyG	8.75 ± 0.65	8.69 ± 0.67	8.80 ± 0.63	< 0.001^∗∗∗^	8.79 ± 0.67	8.71 ± 0.64	< 0.001^∗∗∗^
[M (P25, P75)]	(8.28, 9.13)	(8.20, 9.08)	(8.34, 9.18)		(8.30, 9.19)	(8.26, 9.09)	

*Note:* P25, 25th percentile; P75, 75th percentile; M, median; MetS, metabolic syndrome; TyG, triglyceride glucose. ^∗^*p* < 0.05, ^∗∗^*p* < 0.01, ^∗∗∗^*p* < 0.001, indicating statistically significant differences.

Abbreviations: BMI, body mass index; CRP, C-reactive protein; CVAI, Chinese visceral adiposity index; DBP, diastolic blood pressure; FBG, fasting blood glucose; HDL-C, high-density lipoprotein cholesterol; LAP, lipid accumulation product; SBP, systolic blood pressure; VAI, visceral adiposity index; WC, waist circumference.

**Table 2:** Prevalence of MetS and its components in the LAP, VAI, CVAI, TyG, BMI, and WC quartiles.

Index	Group	IDF		China-2020		NCEP ATP III	
		Yes (4390)	No (6437)	Yes (3092)	No (7548)	Yes (3080)	No (7747)
LAP	Q1	49 (1.81)	2660 (98.19)	66 (2.44)	2643 (97.56)	105 (3.88)	2604 (96.12)
	Q2	547 (20.21)^a^	2158 (79.78)	228 (8.43)^a^	2477 (91.57)	234 (8.65)	2471 (91.35)
	Q3	1376 (50.83)^ab^	1331 (49.17)	814 (30.07)^ab^	1893 (69.93)	825 (30.48)^ab^	1882 (69.52)
	Q4	2418 (89.36)^abc^	288 (10.64)	1984 (73.32)^abc^	722 (26.68)	1916 (70.81)^abc^	790 (29.19)

VAI	Q1	167 (6.17)	2540 (93.83)	80 (2.96)	2627 (97.04)	99 (3.66)	2608 (96.34)
	Q2	610 (22.53)^a^	2097 (77.47)	290 (10.71)	2417 (89.29)	292 (10.79)^a^	2415 (89.21)
	Q3	1366 (50.46)^ab^	1341 (49.54)	838 (30.96)^ab^	1869 (69.04)	861 (31.81)^ab^	1846 (68.19)
	Q4	2247 (83.04)^abc^	459 (16.96)	1884 (69.62)^abc^	822 (30.38)	1828 (67.55)^abc^	878 (32.45)

CVAI	Q1	6 (0.22)	2701 (99.78)	86 (3.18)	2621 (96.82)	132 (4.88)	2575 (95.12)
	Q2	451 (16.66)^a^	2256 (83.34)	341 (12.60)^a^	2366 (87.40)	345 (12.74)^a^	2362 (87.26)
	Q3	1595 (58.92)^ab^	1112 (41.08)	945 (34.91)^ab^	1762 (65.09)	790 (29.18)^ab^	1917 (70.82)
	Q4	2338 (86.37)^abc^	368 (13.60)	1720 (63.56)^abc^	986 (36.44)	1813 (67.00)^abc^	893 (33.00)

TyG	Q1	267 (9.85)	2443 (90.15)	61 (2.25)	2649 (97.75)	104 (3.84)	2606 (96.16)
	Q2	630 (23.25)^a^	2073 (76.69)	204 (7.55)^a^	2499 (92.45)	244 (9.03)^a^	2459 (90.97)
	Q3	1431 (52.80)^ab^	1278 (47.18)	912 (33.67)^ab^	1797 (66.33)	878 (32.41)^ab^	1831 (67.59)
	Q4	2061 (76.05)^abc^	643 (23.78)	1914 (70.78)^abc^	790 (29.22)	1853 (68.53)^abc^	851 (31.47)

BMI	Q1	159 (5.87)	2548 (94.13)	142 (5.25)	2565 (94.75)	193 (7.13)	2514 (92.87)
	Q2	741 (27.37)^a^	1966 (72.63)	472 (17.44)^a^	2235 (82.56)	489 (18.06)^a^	2218 (81.94)
	Q3	1448 (53.49)^ab^	1259 (46.51)	985 (36.39)^ab^	1722 (63.61)	882 (32.58)^ab^	1825 (67.42)
	Q4	2042 (75.43)^abc^	664 (24.54)	1493 (55.17)^abc^	1213 (44.83)	1516 (56.02)^abc^	1190 (43.98)

WC	Q1	0 (0.00)	2723 (100.00)	122 (4.48)	2601 (95.52)	184 (6.76)	2539 (93.24)
	Q2	772 (28.52)^a^	1939 (71.52)	335 (12.36)^a^	2376 (87.64)	377 (13.91)^a^	2334 (86.09)
	Q3	1485 (54.86)^ab^	1206 (44.82)	1057 (39.28)^ab^	1634 (60.72)	977 (36.31)^ab^	1714 (63.69)
	Q4	2133 (78.80)^abc^	569 (21.06)	1578 (58.40)^abc^	1124 (41.60)	1542 (57.07)^abc^	1160 (42.93)

*Note:* MetS, metabolic syndrome; TyG, triglyceride glucose.

Abbreviations: BMI, body mass index; CVAI, Chinese visceral adiposity index; FBG, fasting blood glucose; HDL-C, high-density lipoprotein cholesterol; LAP, lipid accumulation product; VAI, visceral adiposity index; WC, waist circumference.

^a^compared with the Q1 group, *p* < 0.001.

^ab^compared with the Q2 group, *p* < 0.001.

^abc^compared with the Q3 group, *p* < 0.001.

**Table 3:** Cramer's V of MetS and its components in the LAP, VAI, CVAI, TyG, BMI, and WC quartiles.

Index		LAP	VAI	CVAI	TyG	BMI	WC
MetS	IDF	**0.6757**	0.5947	**0.6934**	0.5255	0.5352	**0.6011**
	China-2020	**0.6156**	0.5715	0.515	0.6004	0.4196	0.476
	NCEP ATP III	0.5858	0.5504	0.5302	0.5655	0.4056	0.4383

Elevated waist	IDF	**0.6923**	0.4862	**0.7994**	0.3307	**0.6599**	**0.7597**
	China-2020	**0.6724**	0.4496	**0.7547**	0.3431	**0.7076**	**0.85**
	NCEP ATP III	0.5412	0.3881	**0.703**	0.2386	0.5832	0.6477

Reduced HDL-C	IDF/NCEP ATP III	0.3652	0.5302	0.4222	0.3119	0.2276	0.2133
	China-2020	0.2235	0.3325	0.2029	0.2328	0.1396	0.1658

Elevated fasting glucose	IDF/NCEP ATP III	0.2594	0.2331	0.2118	0.4414	0.1787	0.2052
	China-2020	0.2415	0.2241	0.1900	0.4494	0.1499	0.1769

Elevated triglycerides		**0.7316**	**0.7958**	0.4109	**0.8003**	0.2773	0.2852

Elevated blood pressure		0.1174	0.1174	0.2256	0.1682	0.2164	0.2347

*Note:* MetS, metabolic syndrome; TyG, triglyceride glucose. The significance of bold values indicate strong correlation (≥ 0.6).

Abbreviations: BMI, body mass index; CVAI, Chinese visceral adiposity index; FBG, fasting blood glucose; HDL-C, high-density lipoprotein cholesterol; LAP, lipid accumulation product; VAI, visceral adiposity index; WC, waist circumference.

**Table 4:** Logistic regression analysis of LAP, VAI, CVAI, and TyG parameters for predicting the risk of MetS.

Criterion	Index	Model 1			Model 2			Model 3			Model 4		
		OR (95% CI)	*p* value	AUC	OR (95% CI)	*p* value	AUC	OR (95% CI)	*p* value	AUC	OR (95% CI)	*p* value	AUC
IDF	LAP	6.144 (5.763, 6.551)	< 0.001^∗∗∗^	0.880	6.080 (5.692, 6.494)	< 0.001^∗∗∗^	0.896	6.925 (6.292, 7.622)	< 0.001^∗∗∗^	0.921	6.747 (6.106, 7.457)	< 0.001^∗∗∗^	0.924
	VAI	4.092 (3.883, 4.312)	< 0.001^∗∗∗^	0.835	3.931 (3.726, 4.148)	< 0.001^∗∗∗^	0.848	3.173 (2.892, 3.481)	< 0.001^∗∗∗^	0.878	3.123 (2.831, 3.445)	< 0.001^∗∗∗^	0.884
	CVAI	6.899 (6.445, 4457.384)	< 0.001^∗∗∗^	0.890	8.982 (8.288, 9.734)	< 0.001^∗∗∗^	0.910	6.589 (6.035, 7.193)	< 0.001^∗∗∗^	0.928	6.267 (5.726, 6.858)	< 0.001^∗∗∗^	0.930
	TyG	3.172 (3.028, 3.323)	< 0.001^∗∗∗^	0.796	3.303 (3.145, 3.469)	< 0.001^∗∗∗^	0.829	2.462 (2.264, 2.677)	< 0.001^∗∗∗^	0.871	2.465 (2.255, 2.694)	< 0.001^∗∗∗^	0.879
	BMI	3.330 (3.176, 3.492)	< 0.001^∗∗∗^	0.805	3.481 (3.308, 3.662)	< 0.001^∗∗∗^	0.835	3.065 (2.888, 3.253)	< 0.001^∗∗∗^	0.906	3.002 (2.821, 3.194)	< 0.001^∗∗∗^	0.910
	WC	4.294 (4.070, 4.530)	< 0.001^∗∗∗^	0.842	5.062 (4.765, 5.378)	< 0.001^∗∗∗^	0.877	4.820 (4.487, 5.177)	< 0.001^∗∗∗^	0.932	4.630 (4.301, 4.985)	< 0.001^∗∗∗^	0.933

NCEP ATP III	LAP	4.518 (4.251, 4.802)	< 0.001^∗∗∗^	0.842	4.928 (4.620, 5.256)	< 0.001^∗∗∗^	0.859	3.277 (3.001, 3.578)	< 0.001^∗∗∗^	0.913	3.297 (3.008, 3.613)	< 0.001^∗∗∗^	0.913
	VAI	4.000 (3.776, 4.236)	< 0.001^∗∗∗^	0.827	4.449 (4.182, 4.732)	< 0.001^∗∗∗^	0.844	2.828 (2.547, 3.140)	< 0.001^∗∗∗^	0.901	2.780 (2.494, 3.098)	< 0.001^∗∗∗^	0.900
	CVAI	3.586 (3.396, 3.787)	< 0.001^∗∗∗^	0.811	4.353 (4.091, 4.632)	< 0.001^∗∗∗^	0.830	2.601 (2.414, 2.801)	< 0.001^∗∗∗^	0.911	2.656 (2.457, 2.872)	< 0.001^∗∗∗^	0.911
	TyG	4.232 (3.990, 4.490)	< 0.001^∗∗∗^	0.834	4.389 (4.129, 4.665)	< 0.001^∗∗∗^	0.850	2.956 (2.675, 3.266)	< 0.001^∗∗∗^	0.901	2.956 (2.666, 3.278)	< 0.001^∗∗∗^	0.900
	BMI	2.487 (2.375, 2.605)	< 0.001^∗∗∗^	0.748	2.651 (2.525, 2.784)	< 0.001^∗∗∗^	0.768	2.102 (1.977, 2.235)	< 0.001^∗∗∗^	0.909	2.137 (2.005, 2.278)	< 0.001^∗∗∗^	0.909
	WC	2.730 (2.602, 2.864)	< 0.001^∗∗∗^	0.766	2.800 (2.666, 2.941)	< 0.001^∗∗∗^	0.784	2.234 (2.103, 2.374)	< 0.001^∗∗∗^	0.913	2.247 (2.110, 2.394)	< 0.001^∗∗∗^	0.913

China-2020	LAP	5.282 (4.946, 5.640)	< 0.001^∗∗∗^	0.859	6.078 (5.663, 6.524)	< 0.001^∗∗∗^	0.878	4.402 (3.989, 4.857)	< 0.001^∗∗∗^	0.931	4.617 (4.156, 5.129)	< 0.001^∗∗∗^	0.933
	VAI	4.352 (4.099, 4.620)	< 0.001^∗∗∗^	0.837	5.309 (4.966, 5.676)	< 0.001^∗∗∗^	0.861	3.507 (3.135, 3.923)	< 0.001^∗∗∗^	0.916	3.685 (3.273, 4.148)	< 0.001^∗∗∗^	0.918
	CVAI	3.592 (3.401, 3.793)	< 0.001^∗∗∗^	0.812	5.217 (4.877, 5.581)	< 0.001^∗∗∗^	0.848	3.169 (2.924, 3.434)	< 0.001^∗∗∗^	0.928	3.268 (3.000, 3.559)	< 0.001^∗∗∗^	0.930
	TyG	5.084 (4.766, 5.422)	< 0.001^∗∗∗^	0.855	5.238 (4.904, 5.595)	< 0.001^∗∗∗^	0.864	3.857 (3.458, 4.302)	< 0.001^∗∗∗^	0.917	3.976 (3.547, 4.458)	< 0.001^∗∗∗^	0.919
	BMI	2.627 (2.506, 2.754)	< 0.001^∗∗∗^	0.758	2.792 (2.657, 2.934)	< 0.001^∗∗∗^	0.773	2.304 (2.160, 2.459)	< 0.001^∗∗∗^	0.924	2.324 (2.171, 2.488)	< 0.001^∗∗∗^	0.925
	WC	3.102 (2.949, 3.264)	< 0.001^∗∗∗^	0.789	3.129 (2.973, 3.294)	< 0.001^∗∗∗^	0.796	2.696 (2.522, 2.882)	< 0.001^∗∗∗^	0.931	2.706 (2.523, 2.903)	< 0.001^∗∗∗^	0.932

*Note:* MetS, metabolic syndrome; TyG, triglyceride glucose; AUC, area under the curve; Model 1: unadjusted model. Model 2: Model 1+ age, sex, marital status, education level, smoking status, and alcohol consumption status. Model 3: Model 2 + SBP, DBP, TG, HDL-C, and FBG. Model 4: Model 3 + place of residence, social activities, and health insurance.

Abbreviations: BMI, body mass index; CVAI, Chinese visceral adiposity index; LAP, lipid accumulation product; VAI, visceral adiposity index; WC, waist circumference.

^∗∗∗^ *p* < 0.001 (extremely significant).

**Table 5:** ROC curves of six body fat metrics for predicting the risk of MetS.

Criterion	Index	AUC (95% CI)	*p* value	Sensitivity (%)	Specificity (%)	LR+	LR−	Threshold
IDF	LAP	**0.903 (0.898, 0.909)**	< 0.001^∗∗∗^	81.39	81.67	4.440	0.228	37.63
	VAI	0.854 (0.846, 0.861)	< 0.001^∗∗∗^	78.18	77.41	3.461	0.282	4.20
	CVAI	**0.909 (0.904, 0.914)**	< 0.001^∗∗∗^	90.36	76.53	3.850	0.126	147.60
	TyG	0.815 (0.807, 0.823)	< 0.001^∗∗∗^	73.46	77.75	3.302	0.341	8.78
	BMI	0.822 (0.814, 0.830)	< 0.001^∗∗∗^	77.18	73.00	2.858	0.313	24.00
	WC	0.860 (0.853, 0.867)	< 0.001^∗∗∗^	72.65	84.71	4.752	0.323	89.40

NCEP ATP III	LAP	**0.862 (0.854, 0.870)**	< 0.001^∗∗∗^	80.84	77.76	3.635	0.246	42.01
	VAI	0.846 (0.838, 0.854)	< 0.001^∗∗∗^	79.77	75.29	3.229	0.269	4.59
	CVAI	0.832 (0.823, 0.840)	< 0.001^∗∗∗^	75.00	76.67	3.215	0.326	162.30
	TyG	**0.856 (0.848, 0.863)**	< 0.001^∗∗∗^	84.54	79.44	4.113	0.195	8.85
	BMI	0.765 (0.755, 0.775)	< 0.001^∗∗∗^	70.03	70.89	2.406	0.423	24.65
	WC	0.786 (0.776, 0.795)	< 0.001^∗∗∗^	78.83	68.17	2.477	0.311	87.70

China-2020	LAP	**0.881 (0.874, 0.888)**	< 0.001^∗∗∗^	80.14	81.65	4.369	0.243	44.76
	VAI	0.860 (0.852, 0.867)	< 0.001^∗∗∗^	80.82	76.81	3.485	0.250	4.66
	CVAI	0.831 (0.823, 8230.839)	< 0.001^∗∗∗^	84.09	66.80	2.533	0.238	150.90
	TyG	**0.879 (0.872, 0.886)**	< 0.001^∗∗∗^	84.54	79.44	4.113	0.195	8.85
	BMI	0.775 (0.766, 0.784)	< 0.001^∗∗∗^	77.26	66.13	2.281	0.344	23.42
	WC	0.808 (0.799, 0.816)	< 0.001^∗∗∗^	79.33	70.92	2.729	0.291	88.10

*Note:* AUC, area under the curve; LR+: positive likelihood ratio; LR−: negative likelihood ratio. The significance of the bold values are the top two values of this standard.

^∗∗∗^ *p* < 0.001 (extremely significant)

**Table 6:** Pairwise comparisons of the AUCs of six body fat metrics in predicting MetS by the DeLong test.

Criterion	Variable	LAP	VAI	CVAI	TyG	BMI
IDF	VAI	22.594^∗∗∗^				
	CVAI	1.991^∗^	14.833^∗∗∗^			
	TyG	31.636^∗∗∗^	14.604^∗∗∗^	20.666^∗∗∗^		
	BMI	20.7^∗∗∗^	6.065^∗∗∗^	25.774^∗∗∗^	1.303	
	WC	13.137^∗∗∗^	1.295	17.251^∗∗∗^	8.641^∗∗∗^	13.149^∗∗∗^

NCEP ATP III	VAI	5.679^∗∗∗^				
	CVAI	8.844^∗∗∗^	3.246^∗^			
	TyG	1.803	3.398^∗∗^	4.457^∗∗∗^		
	BMI	19.972^∗∗∗^	13.501^∗∗∗^	16.298^∗∗∗^	14.706^∗∗∗^	
	WC	19.073^∗∗∗^	10.693^∗∗∗^	13.252^∗∗∗^	11.518^∗∗∗^	5.845^∗∗∗^

China-2020	VAI	8.336^∗∗∗^				
	CVAI	15.056^∗∗∗^	6.803^∗∗∗^			
	TyG	0.787	6.864^∗∗∗^	9.652^∗∗∗^		
	BMI	22.595^∗∗∗^	14.644^∗∗∗^	13.919^∗∗∗^	18.058^∗∗∗^	
	WC	18.825^∗∗∗^	9.563^∗∗∗^	6.736^∗∗∗^	12.598^∗∗∗^	9.49^∗∗∗^

^∗^ *p* < 0.05.

^∗∗^ *p* < 0.01.

^∗∗∗^ *p* < 0.001.

## Data Availability

The datasets used and/or analyzed during the current study are available from the corresponding author upon reasonable request.

## Nomenclature

CHARLS: China Health and Retirement Longitudinal Study
AUC: Area under the curve
BMI: Body mass index
LAP: Lipid accumulation product
CVAI: Chinese visceral adiposity index
MetS: Metabolic syndrome
TyG: Triglyceride glucose
VAI: Visceral adiposity index
LR+: Positive likelihood ratio;
LR−: Negative likelihood ratio

## Supporting Information

This supporting information provides additional analyses comparing body fat metrics in predicting MetS. Figure S1 shows a Directed Acyclic Graph of causal relationships. Tables S1–S4 present logistic regression analyses of LAP, VAI, CVAI, and TyG indices. Tables S5–S8 provide ROC analyses with AUC values and optimal cutoffs. Tables S9–S12 show DeLong test results for pairwise AUC comparisons. All analyses are stratified by sex and age (< 60/≥ 60 years).

## References

[1] Fahed G., Aoun L., Bou Zerdan M. (2022). Metabolic Syndrome: Updates on Pathophysiology and Management in 2021. *IJMS*.

[2] Noubiap J. J., Nansseu J. R., Lontchi-Yimagou E. (2022). Geographic Distribution of Metabolic Syndrome and Its Components in the General Adult Population: A Meta-Analysis of Global Data From 28 Million Individuals. *Diabetes Research and Clinical Practice*.

[3] Hsu C.-N., Hou C.-Y., Hsu W.-H., Tain Y.-L. (2021). Early-Life Origins of Metabolic Syndrome: Mechanisms and Preventive Aspects. *IJMS*.

[4] Tune J. D., Goodwill A. G., Sassoon D. J., Mather K. J. (2017). Cardiovascular Consequences of Metabolic Syndrome. *Translational Research*.

[5] Amouzegar A., Honarvar M., Masoumi S., Khalili D., Azizi F., Mehran L. (2023). Trajectory Patterns of Metabolic Syndrome Severity Score and Risk of Type 2 Diabetes. *Journal of Translational Medicine*.

[6] Ahn H.-J., Lee S.-R., Choi E.-K. (2024). Metabolic Syndrome and Ischaemic Stroke in Non-Anticoagulated Atrial Fibrillation With Low CHA_2_ DS_2_-VASc Scores. *Heart*.

[7] Prasun P. (2020). Mitochondrial Dysfunction in Metabolic Syndrome. *Biochimica et Biophysica Acta (BBA)—Molecular Basis of Disease*.

[8] Pammer L. M., Lamina C., Schultheiss U. T. (2021). Association of the Metabolic Syndrome With Mortality and Major Adverse Cardiac Events: A Large Chronic Kidney Disease Cohort. *Journal of Internal Medicine*.

[9] Saklayen M. G. (2018). The Global Epidemic of the Metabolic Syndrome. *Current Hypertension Reports*.

[10] Chen H., Zheng X., Zong X. (2021). Metabolic Syndrome, Metabolic Comorbid Conditions and Risk of Early-Onset Colorectal Cancer. *Gut*.

[11] Borga M., West J., Bell J. D. (2018). Advanced Body Composition Assessment: From Body Mass Index to Body Composition Profiling. *Journal of Investigative Medicine*.

[12] Pickhardt P. J., Graffy P. M., Zea R. (2021). Utilizing Fully Automated Abdominal CT–Based Biomarkers for Opportunistic Screening for Metabolic Syndrome in Adults Without Symptoms. *American Journal of Roentgenology*.

[13] Lee W. (2016). Body Fatness Charts Based on BMI and Waist Circumference. *Obesity*.

[14] Moltrer M., Pala L., Cosentino C., Mannucci E., Rotella C. M., Cresci B. (2022). Body Mass Index (BMI), Waist Circumference (WC), Waist-to-Height Ratio (Whtr) E Waist Body Mass Index (Wbmi): Which is Better?. *Endocrine*.

[15] Kang S. W., Kim S. K., Kim Y. S., Park M.-S. (2023). Risk Prediction of the Metabolic Syndrome Using Tyg Index and Snps: A 10-Year Longitudinal Prospective Cohort Study. *Molecular and Cellular Biochemistry*.

[16] Nabipoorashrafi S. A., Seyedi S. A., Rabizadeh S. (2022). The Accuracy of Triglyceride-Glucose (Tyg) Index for the Screening of Metabolic Syndrome in Adults: A Systematic Review and Meta-Analysis. *Nutrition, Metabolism, and Cardiovascular Diseases*.

[17] Li Y., Zheng R., Li S. (2022). Association Between Four Anthropometric Indexes and Metabolic Syndrome in US Adults. *Frontiers in Endocrinology*.

[18] Adil S. O., Musa K. I., Uddin F., Shafique K., Khan A., Islam M. A. (2023). Role of Anthropometric Indices as a Screening Tool for Predicting Metabolic Syndrome Among Apparently Healthy Individuals of Karachi, Pakistan. *Frontiers in Endocrinology*.

[19] Liu X., Ma C., Yin F. (2021). Performance of Two Novel Obesity Indicators for the Management of Metabolic Syndrome in Young Adults. *Frontiers in Endocrinology*.

[20] Xia M.-F., Chen Y., Lin H.-D. (2016). A Indicator of Visceral Adipose Dysfunction to Evaluate Metabolic Health in Adult Chinese. *Scientific Reports*.

[21] Xia M., Lin H., Chen L. (2018). Association of Visceral Adiposity and its Longitudinal Increase With the Risk of Diabetes in Chinese Adults: A Prospective Cohort Study. *Diabetes Metabolism Research*.

[22] Han M., Qin P., Li Q. (2021). Chinese Visceral Adiposity Index: A Reliable Indicator of Visceral Fat Function Associated With Risk of Type 2 Diabetes. *Diabetes Metabolism Research*.

[23] Lear S. A., Humphries K. H., Kohli S., Birmingham C. L. (2007). The Use of BMI and Waist Circumference as Surrogates of Body Fat Differs by Ethnicity. *Obesity*.

[24] Jones D. W. (2019). Implementing Automated Office Blood Pressure Measurement: Controversies in Hypertension–Pro Side of the Argument. *Hypertension*.

[25] Fahmy A. M., El Shall N., Kabbash I., El Ahwal L., Selim A. (2023). Lipid Accumulation Product and Visceral Adiposity Index: Two Indices to Predict Metabolic Syndrome and Insulin Resistance in Chronic Kidney Disease Patients. *Endocrine Regulations*.

[26] Sergi G., Dianin M., Bertocco A. (2020). Gender Differences in the Impact of Metabolic Syndrome Components on Mortality in Older People: A Systematic Review and Meta-Analysis. *Nutrition, Metabolism, and Cardiovascular Diseases*.

[27] Masrouri S., Shapiro M. D., Khalili D., Hadaegh F. (2024). Impact of Coronary Artery Calcium on Mortality and Cardiovascular Events in Metabolic Syndrome and Diabetes Among Younger Adults. *European Journal of Preventive Cardiology*.

[28] Li Y., Gui J., Liu H. (2023). Predicting Metabolic Syndrome by Obesity- and Lipid-Related Indices in Mid-Aged and Elderly Chinese: A Population-Based Cross-Sectional Study. *Frontiers in Endocrinology*.

[29] Huang Y., Zhang L., Wang Z. (2022). The Prevalence and Characteristics of Metabolic Syndrome According to Different Definitions in China: A Nationwide Cross-Sectional Study, 2012–2015. *BMC Public Health*.

[30] Li W., Chen D., Peng Y., Lu Z., Kwan M.-P., Tse L. A. (2023). Association Between Metabolic Syndrome and Mortality: Prospective Cohort Study. *JMIR Public Health Surveillance*.

[31] Yu S. R., Shin K.-A. (2023). Visceral Adiposity Index and Lipid Accumulation Product as Effective Markers of Different Obesity Phenotypes in Korean Adults: A Cross-Sectional Analysis. *DMSO*.

[32] Naghshband Z., Kumar L., Mandappa S., Niranjana Murthy A., Malini S. (2021). Visceral Adiposity Index and Lipid Accumulation Product as Diagnostic Markers of Metabolic Syndrome in South Indians With Polycystic Ovary Syndrome. *Journal of Human Reproductive Sciences*.

[33] Mohamed W., Ishak K., Baharum N. (2024). Ethnic Disparities and its Association Between Epicardial Adipose Tissue Thickness and Cardiometabolic Parameters. *Adipocyte*.

[34] Chen J., Li Y. T., Niu Z. (2024). Association of Visceral Obesity Indices With Incident Diabetic Retinopathy in Patients With Diabetes: Prospective Cohort Study. *JMIR Public Health Surveillance*.

[35] Khan S. H., Sobia F., Niazi N. K., Manzoor S. M., Fazal N., Ahmad F. (2018). Metabolic Clustering of Risk Factors: Evaluation of Triglyceride-Glucose Index (Tyg Index) for Evaluation of Insulin Resistance. *Diabetology & Metabolic Syndrome*.

[36] Mauvais-Jarvis F. (2024). Sex Differences in Energy Metabolism: Natural Selection, Mechanisms and Consequences. *Nature Reviews Nephrology*.

[37] Palacios-González B., León-Reyes G., Rivera-Paredez B. (2022). Targeted Metabolomics Revealed a Sex-Dependent Signature for Metabolic Syndrome in the Mexican Population. *Nutrients*.

[38] Palmer B. F., Clegg D. J. (2015). The Sexual Dimorphism of Obesity. *Molecular and Cellular Endocrinology*.

[39] Kautzky-Willer A., Harreiter J., Pacini G. (2016). Sex and Gender Differences in Risk, Pathophysiology and Complications of Type 2 Diabetes Mellitus. *Endocrine Reviews*.

[40] Arnetz L., Ekberg N. R., Alvarsson M. (2014). Sex Differences in Type 2 Diabetes: Focus on Disease Course and Outcomes. *Diabetes, Metabolic Syndrome and Obesity: Targets and Therapy*.

[41] Mosad A., Elfadil G., Elhassan S. (2023). Diagnostic Performance Using Obesity and Lipid-Related Indices and Atherogenic Index of Plasma to Predict Metabolic Syndrome in the Adult Sudanese Population. *Nigerian Journal of Clinical Practice*.

[42] Li H., Zhang Y., Luo H., Lin R. (2022). The Lipid Accumulation Product is a Powerful Tool to Diagnose Metabolic Dysfunction-Associated Fatty Liver Disease in the United States Adults. *Frontiers in Endocrinology*.

[43] Alfawaz H. A., Khan N., Ansari M. G. A., Khattak M. N. K., Saadawy G. M., Al-Daghri N. M. (2023). Sex-Specific Cut-Offs of Seven Adiposity Indicators and Their Performance in Predicting Metabolic Syndrome in Arab Adults. *JCM*.

[44] Al-Sofiani M. E., Ganji S. S., Kalyani R. R. (2019). Body Composition Changes in Diabetes and Aging. *Journal of Diabetes and its Complications*.

[45] Buchmann N., Ittermann T., Demuth I. (2022). Lipoprotein(A) and Metabolic Syndrome. *Deutsches Ärzteblatt international*.

[46] Harris T. B. (2017). Weight and Body Mass Index in Old Age: Do They Still Matter?. *Journal of the American Geriatrics Society*.

[47] Greendale G. A., Han W., Finkelstein J. S. (2021). Changes in Regional Fat Distribution and Anthropometric Measures Across the Menopause Transition. *Journal of Clinical Endocrinology and Metabolism*.

[48] Ambikairajah A., Walsh E., Tabatabaei-Jafari H., Cherbuin N. (2019). Fat Mass Changes During Menopause: A Metaanalysis. *American Journal of Obstetrics and Gynecology*.

[49] Dehghan A., Vasan S. K., Fielding B. A., Karpe F. (2021). A Prospective Study of the Relationships Between Change in Body Composition and Cardiovascular Risk Factors Across the Menopause. *Menopause*.

