# Supplementary material for: Precision Screening for MetS: The Role of Derived Lipid Indicators in Chinese Populations
Source: Int J Endocrinol. 2025 Oct 29;2025:9990629. doi: 10.1155/ije/9990629 (PMC12588751; doi:10.1155/ije/9990629)
Supplement: Supporting Information — Additional supporting information can be found online in the Supporting Information section. [file 9990629.f1.docx]

|  | ***Supplementary Material* Contents** |
| --- | --- |
| No. | **Precision Screening for MetS: The Role of Derived Lipid Indicators in Chinese Populations** |
|  | Jiayu Zhou^a1^, Weifang Dai^b1^, Weina Xu^c^, Shanna Liu^b^, Qingli Zhou^b⁎^ ^a^ School of Medicine，Shihezi University，Shihezi，Xinjiang 832000，China. ^B^ Department of Information Technology, the Fourth Affiliated Hospital of School of Medicine, and International School of Medicine, International Institutes of Medicine，Zhejiang University, Yiwu，China , 322000. ^c^ Department of Geriatric, Center for Regeneration and Aging Medicine，the Fourth Affiliated Hospital of School of Medicine, and International School of Medicine, International Institutes of Medicine，Zhejiang University, Yiwu，China , 322000.  1These authors contributed equally to this work and should be considered co-first authors ⁎ Corresponding authors: zhouql@zju.edu.cn. |
| 1 | Figure S1. Directed Acyclic Graph(DAG) for Metabolic Syndrome |
| 2 | Table S1. Logistic regression analysis of LAP, VAI, CVAI, and TyG parameters predicting the risk of MetS (in male) |
| 3 | Table S2. Logistic regression analysis of LAP, VAI, CVAI, and TyG parameters predicting the risk of MetS (in female) |
| 4 | Table S3. Logistic regression analysis of LAP, VAI, CVAI, and TyG parameters predicting the risk of MetS (in age<60) |
| 5 | Table S4. Logistic regression analysis of LAP, VAI, CVAI, and TyG parameters predicting the risk of MetS (in age>=60) |
| 6 | Table S5. ROC analysis of six body fat metrics to predict the risk of MetS (in male) |
| 7 | Table S6. ROC analysis of six body fat metrics to predict the risk of MetS (in female) |
| 8 | Table S7. ROC analysis of six body fat metrics to predict the risk of MetS (in age<60) |
| 9 | Table S8. ROC analysis of six body fat metrics to predict the risk of MetS (in age>=60) |
| 10 | Table S9. Pairwise comparisons of the AUCs of six body fat metrics in predicting Mets by the Delong test.(in male) |
| 11 | Table S10. Pairwise comparisons of the AUCs of six body fat metrics in predicting Mets by the Delong test.(in female) |
| 12 | Table S11. Pairwise comparisons of the AUCs of six body fat metrics in predicting Mets by the Delong test. (in age<60) |
| 13 | Table S12. Pairwise comparisons of the AUCs of six body fat metrics in predicting Mets by the Delong test.(in age>=60) |


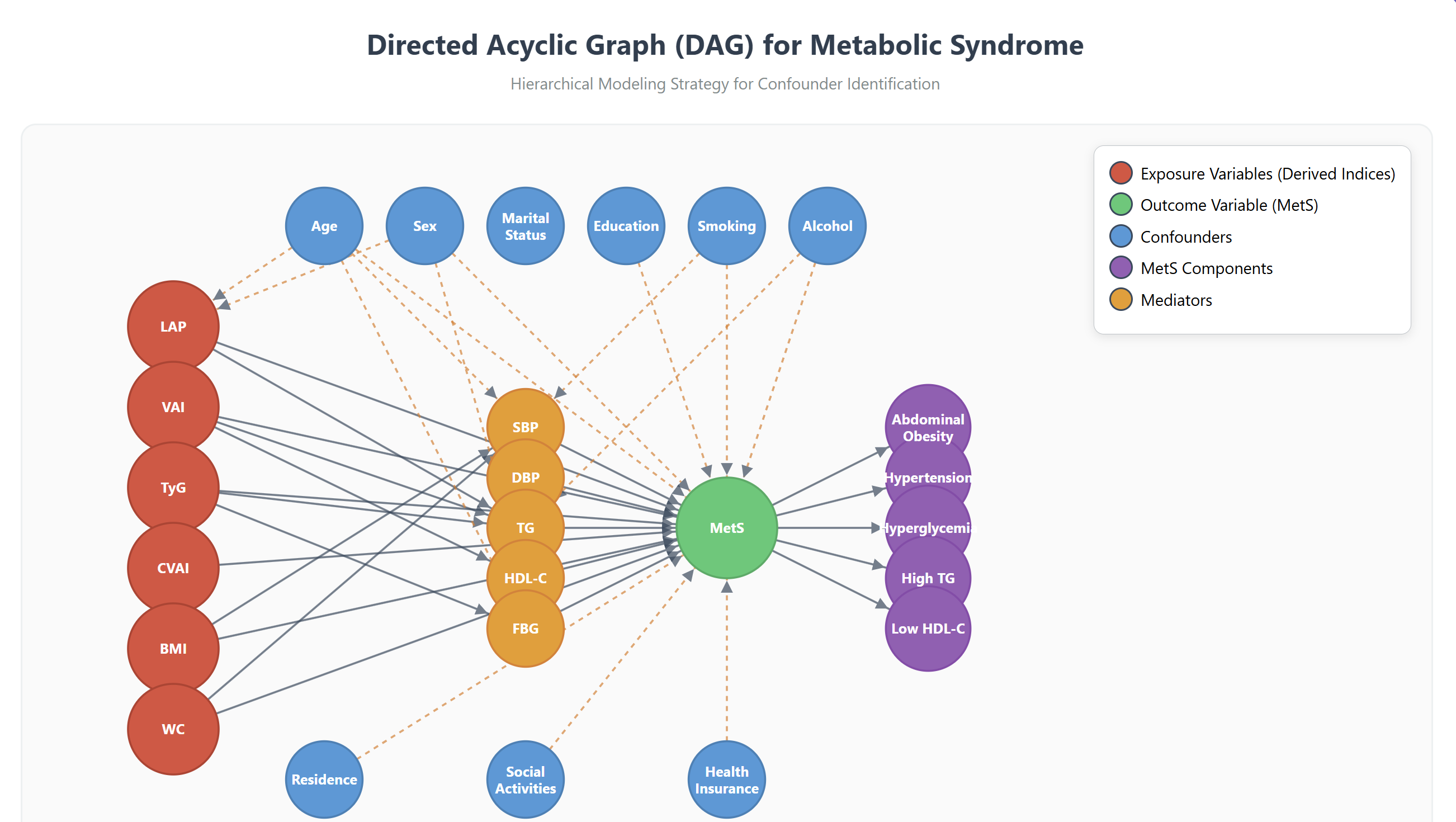


Figure S1. Directed Acyclic Graph(DAG) for Metabolic Syndrome

| Table S1　Logistic regression analysis of LAP, VAI, CVAI, and TyG parameters predicting the risk of MetS (in male) | | | | | | | | | | | | |  |
| --- | --- | --- | --- | --- | --- | --- | --- | --- | --- | --- | --- | --- | --- |
| criterion | index | model1 | | | model2 | | | model3 | | | model4 | | |
|  |  | OR(95%CI) | *P-value* | AUC | OR(95%CI) | *P-value* | AUC | OR(95%CI) | *P-value* | AUC | OR(95%CI) | *P-value* | AUC |
| IDF | LAP | 6.684(6.031 ,7.409) | <0.001*** | 0.901 | 6.675(6.007 ,7.417) | <0.001*** | 0.908 | 10.199(8.662 ,12.008) | <0.001*** | 0.930 | 10.487(8.801 ,12.496) | <0.001*** | 0.934 |
|  | VAI | 3.549(3.294 ,3.824) | <0.001*** | 0.824 | 3.506(3.249 ,3.783) | <0.001*** | 0.841 | 3.107(2.699 ,3.575) | <0.001*** | 0.864 | 3.162(2.716 ,3.680) | <0.001*** | 0.873 |
|  | CVAI | 10.435(9.198 ,11.839) | <0.001*** | 0.933 | 11.521(10.068 ,13.184) | <0.001*** | 0.948 | 10.508(9.030 ,12.229) | <0.001*** | 0.954 | 9.928(8.488 ,11.613) | <0.001*** | 0.956 |
|  | TyG | 3.271(3.037 ,3.523) | <0.001*** | 0.807 | 3.238(3.001 ,3.493) | <0.001*** | 0.826 | 2.529(2.243 ,2.852) | <0.001*** | 0.860 | 2.566(2.254 ,2.920) | <0.001*** | 0.869 |
|  | BMI | 5.621(5.101 ,6.195) | <0.001*** | 0.875 | 5.605(5.075 ,6.191) | <0.001*** | 0.881 | 5.044(4.506 ,5.647) | <0.001*** | 0.924 | 5.013(4.448 ,5.651) | <0.001*** | 0.929 |
|  | WC | 11.490(10.012 ,13.187) | <0.001*** | 0.919 | 11.295(9.832 ,12.976) | <0.001*** | 0.926 | 11.363(9.674 ,13.346) | <0.001*** | 0.956 | 10.987(9.276 ,13.013) | <0.001*** | 0.957 |
| NCEP ATPIII | LAP | 4.140(3.807 ,4.502) | <0.001*** | 0.846 | 4.414(4.038 ,4.825) | <0.001*** | 0.855 | 2.585(2.277 ,2.934) | <0.001*** | 0.920 | 2.575(2.252 ,2.943) | <0.001*** | 0.919 |
|  | VAI | 4.483(4.110 ,4.889) | <0.001*** | 0.856 | 4.683(4.279 ,5.126) | <0.001*** | 0.865 | 2.784(2.356 ,3.290) | <0.001*** | 0.914 | 2.699(2.264 ,3.218) | <0.001*** | 0.913 |
|  | CVAI | 3.484(3.230 ,3.758) | <0.001*** | 0.818 | 3.536(3.272 ,3.821) | <0.001*** | 0.824 | 2.075(1.885 ,2.284) | <0.001*** | 0.922 | 2.114(1.910 ,2.340) | <0.001*** | 0.922 |
|  | TyG | 4.518(4.121 ,4.954) | <0.001*** | 0.849 | 4.705(4.276 ,5.177) | <0.001*** | 0.858 | 2.900(2.482 ,3.388) | <0.001*** | 0.914 | 2.897(2.460 ,3.412) | <0.001*** | 0.913 |
|  | BMI | 2.523(2.353 ,2.706) | <0.001*** | 0.754 | 2.568(2.388 ,2.761) | <0.001*** | 0.759 | 1.781(1.623 ,1.954) | <0.001*** | 0.917 | 1.795(1.629 ,1.979) | <0.001*** | 0.916 |
|  | WC | 2.595(2.416 ,2.788) | <0.001*** | 0.759 | 2.595(2.412 ,2.792) | <0.001*** | 0.761 | 1.727(1.576 ,1.893) | <0.001*** | 0.917 | 1.731(1.573 ,1.905) | <0.001*** | 0.916 |
| China-2020 | LAP | 5.454(4.970 ,5.984) | <0.001*** | 0.880 | 5.697(5.170 ,6.278) | <0.001*** | 0.887 | 4.208(3.648 ,4.854) | <0.001*** | 0.940 | 4.242(3.644 ,4.938) | <0.001*** | 0.940 |
|  | VAI | 5.000(4.577 ,5.463) | <0.001*** | 0.869 | 5.080(4.639 ,5.562) | <0.001*** | 0.877 | 3.338(2.804 ,3.974) | <0.001*** | 0.925 | 3.411(2.835 ,4.103) | <0.001*** | 0.925 |
|  | CVAI | 4.530(4.168 ,4.924) | <0.001*** | 0.861 | 4.657(4.273 ,5.074) | <0.001*** | 0.872 | 3.033(2.730 ,3.369) | <0.001*** | 0.942 | 3.011(2.696 ,3.364) | <0.001*** | 0.943 |
|  | TyG | 4.957(4.522 ,5.433) | <0.001*** | 0.862 | 5.056(4.601 ,5.558) | <0.001*** | 0.871 | 3.575(3.042 ,4.202) | <0.001*** | 0.926 | 3.695(3.113 ,4.385) | <0.001*** | 0.927 |
|  | BMI | 3.051(2.838 ,3.281) | <0.001*** | 0.792 | 3.080(2.857 ,3.320) | <0.001*** | 0.797 | 2.382(2.159 ,2.628) | <0.001*** | 0.935 | 2.349(2.118 ,2.605) | <0.001*** | 0.934 |
|  | WC | 3.640(3.360 ,3.943) | <0.001*** | 0.819 | 3.614(3.333 ,3.920) | <0.001*** | 0.823 | 2.938(2.643 ,3.265) | <0.001*** | 0.942 | 2.880(2.578 ,3.217) | <0.001*** | 0.942 |
| *Notes: BMI, body mass index; CVAI, Chinese visceral adiposity index; LAP, lipid accumulation product; MetS, metabolic syndrome; TyG, triglyceride glucose; VAI, visceral adiposity index; WC, waist circumference; AUC, area under the curve;***:p<0.001(extreme significant) Model 1: Unadjusted model  Model 2: Model 1+ age, gender, marital status, education level, smoking, and drinking  Model 3: Model 2 + SBP, DBP, TG, HDL-C, and FBG Model 4: Model 3 + place of residence, social activities, and health insurance* | | | | | | | | | | | | |  |

| **Table S2**　Logistic regression analysis of LAP, VAI, CVAI, and TyG parameters predicting the risk of MetS (in female) | | | | | | | | | | | | |  |
| --- | --- | --- | --- | --- | --- | --- | --- | --- | --- | --- | --- | --- | --- |
| criterion | index | model1 | | | model2 | | | model3 | | | model4 | | |
|  |  | OR(95%CI) | *P-value* | AUC | OR(95%CI) | *P-value* | AUC | OR(95%CI) | *P-value* | AUC | OR(95%CI) | *P-value* | AUC |
| IDF | LAP | 5.579(5.126 ,6.072) | <0.001*** | 0.855 | 5.612(5.154 ,6.112) | <0.001*** | 0.863 | 5.543(4.912 ,6.254) | <0.001*** | 0.910 | 5.299(4.680 ,6.000) | <0.001*** | 0.911 |
|  | VAI | 4.268(3.958 ,4.603) | <0.001*** | 0.822 | 4.287(3.973 ,4.625) | <0.001*** | 0.828 | 3.277(2.887 ,3.719) | <0.001*** | 0.877 | 3.154(2.764 ,3.598) | <0.001*** | 0.880 |
|  | CVAI | 5.777(5.287 ,6.314) | <0.001*** | 0.833 | 7.591(6.850 ,8.413) | <0.001*** | 0.860 | 4.927(4.388 ,5.533) | <0.001*** | 0.904 | 4.725(4.195 ,5.322) | <0.001*** | 0.906 |
|  | TyG | 3.320(3.111 ,3.542) | <0.001*** | 0.800 | 3.303(3.095 ,3.524) | <0.001*** | 0.804 | 2.300(2.032 ,2.603) | <0.001*** | 0.865 | 2.303(2.025 ,2.618) | <0.001*** | 0.870 |
|  | BMI | 2.577(2.432 ,2.729) | <0.001*** | 0.753 | 2.753(2.591 ,2.925) | <0.001*** | 0.772 | 2.416(2.245 ,2.600) | <0.001*** | 0.892 | 2.367(2.194 ,2.553) | <0.001*** | 0.895 |
|  | WC | 3.630(3.394 ,3.881) | <0.001*** | 0.814 | 3.641(3.404 ,3.895) | <0.001*** | 0.823 | 3.549(3.267 ,3.856) | <0.001*** | 0.916 | 3.435(3.155 ,3.741) | <0.001*** | 0.916 |
| NCEP ATPIII | LAP | 5.096(4.656 ,5.578) | <0.001*** | 0.831 | 5.420(4.934 ,5.954) | <0.001*** | 0.855 | 4.054(3.574 ,4.598) | <0.001*** | 0.910 | 4.100(3.598 ,4.673) | <0.001*** | 0.910 |
|  | VAI | 4.009(3.689 ,4.357) | <0.001*** | 0.797 | 4.146(3.806 ,4.516) | <0.001*** | 0.819 | 2.844(2.475 ,3.267) | <0.001*** | 0.891 | 2.834(2.456 ,3.271) | <0.001*** | 0.890 |
|  | CVAI | 5.539(5.019 ,6.112) | <0.001*** | 0.812 | 5.960(5.367 ,6.618) | <0.001*** | 0.822 | 3.604(3.186 ,4.077) | <0.001*** | 0.905 | 3.717(3.268 ,4.228) | <0.001*** | 0.905 |
|  | TyG | 4.016(3.716 ,4.342) | <0.001*** | 0.819 | 4.104(3.791 ,4.444) | <0.001*** | 0.837 | 3.108(2.721 ,3.551) | <0.001*** | 0.892 | 3.072(2.681 ,3.519) | <0.001*** | 0.891 |
|  | BMI | 2.409(2.264 ,2.563) | <0.001*** | 0.735 | 2.727(2.550 ,2.915) | <0.001*** | 0.772 | 2.352(2.164 ,2.556) | <0.001*** | 0.904 | 2.403(2.204 ,2.620) | <0.001*** | 0.905 |
|  | WC | 2.879(2.696 ,3.074) | <0.001*** | 0.773 | 2.941(2.751 ,3.144) | <0.001*** | 0.793 | 2.664(2.451 ,2.895) | <0.001*** | 0.913 | 2.683(2.461 ,2.925) | <0.001*** | 0.913 |
| China-2020 | LAP | 6.121(5.534 ,6.771) | <0.001*** | 0.846 | 6.399(5.768 ,7.099) | <0.001*** | 0.865 | 4.558(3.967 ,5.237) | <0.001*** | 0.924 | 4.982(4.289 ,5.786) | <0.001*** | 0.927 |
|  | VAI | 5.303(4.812 ,5.844) | <0.001*** | 0.825 | 5.448(4.934 ,6.015) | <0.001*** | 0.842 | 3.697(3.163 ,4.320) | <0.001*** | 0.910 | 4.055(3.434 ,4.789) | <0.001*** | 0.912 |
|  | CVAI | 5.482(4.952 ,6.068) | <0.001*** | 0.807 | 6.164(5.524 ,6.878) | <0.001*** | 0.822 | 3.448(3.024 ,3.932) | <0.001*** | 0.916 | 3.777(3.278 ,4.351) | <0.001*** | 0.919 |
|  | TyG | 5.241(4.786 ,5.739) | <0.001*** | 0.848 | 5.321(4.853 ,5.835) | <0.001*** | 0.861 | 4.119(3.544 ,4.788) | <0.001*** | 0.911 | 4.234(3.621 ,4.951) | <0.001*** | 0.913 |
|  | BMI | 2.348(2.204 ,2.501) | <0.001*** | 0.727 | 2.564(2.399 ,2.741) | <0.001*** | 0.757 | 2.218(2.034 ,2.418) | <0.001*** | 0.915 | 2.284(2.084 ,2.502) | <0.001*** | 0.917 |
|  | WC | 2.739(2.564 ,2.926) | <0.001*** | 0.762 | 2.763(2.584 ,2.953) | <0.001*** | 0.778 | 2.491(2.285 ,2.716) | <0.001*** | 0.922 | 2.547(2.324 ,2.790) | <0.001*** | 0.924 |
| *Notes: BMI, body mass index; CVAI, Chinese visceral adiposity index; LAP, lipid accumulation product; MetS, metabolic syndrome; TyG, triglyceride glucose; VAI, visceral adiposity index; WC, waist circumference; AUC, area under the curve;***:p<0.001(extreme significant) Model 1: Unadjusted model  Model 2: Model 1+ age, gender, marital status, education level, smoking, and drinking  Model 3: Model 2 + SBP, DBP, TG, HDL-C, and FBG Model 4: Model 3 + place of residence, social activities, and health insurance* | | | | | | | | | | | | |  |

| **Table S3**　Logistic regression analysis of LAP, VAI, CVAI, and TyG parameters predicting the risk of MetS (in age<60) | | | | | | | | | | | | |  |
| --- | --- | --- | --- | --- | --- | --- | --- | --- | --- | --- | --- | --- | --- |
| criterion | index | model1 | | | model2 | | | model3 | | | model4 | | |
|  |  | OR(95%CI) | *P-value* | AUC | OR(95%CI) | *P-value* | AUC | OR(95%CI) | *P-value* | AUC | OR(95%CI) | *P-value* | AUC |
| IDF | LAP | 5.832(5.338 ,6.372) | <0.001*** | 0.872 | 5.889(5.378 ,6.448) | <0.001*** | 0.886 | 6.393(5.602 ,7.295) | <0.001*** | 0.916 | 6.166(5.362 ,7.091) | <0.001*** | 0.921 |
|  | VAI | 4.058(3.768 ,4.370) | <0.001*** | 0.830 | 3.999(3.708 ,4.312) | <0.001*** | 0.841 | 3.185(2.798 ,3.625) | <0.001*** | 0.876 | 3.137(2.727 ,3.608) | <0.001*** | 0.885 |
|  | CVAI | 7.283(6.593 ,8.046) | <0.001*** | 0.883 | 8.035(7.226 ,8.935) | <0.001*** | 0.898 | 5.769(5.132 ,6.485) | <0.001*** | 0.921 | 5.391(4.773 ,6.089) | <0.001*** | 0.925 |
|  | TyG | 3.172(2.971 ,3.387) | <0.001*** | 0.796 | 3.437(3.205 ,3.686) | <0.001*** | 0.826 | 2.534(2.253 ,2.849) | <0.001*** | 0.870 | 2.601(2.289 ,2.955) | <0.001*** | 0.881 |
|  | BMI | 3.339(3.118 ,3.576) | <0.001*** | 0.796 | 3.340(3.114 ,3.582) | <0.001*** | 0.814 | 2.952(2.716 ,3.208) | <0.001*** | 0.901 | 2.848(2.608 ,3.111) | <0.001*** | 0.907 |
|  | WC | 4.103(3.810 ,4.420) | <0.001*** | 0.832 | 4.730(4.358 ,5.135) | <0.001*** | 0.857 | 4.562(4.134 ,5.035) | <0.001*** | 0.926 | 4.317(3.894 ,4.786) | <0.001*** | 0.929 |
| NCEP ATPIII | LAP | 4.499(4.109 ,4.927) | <0.001*** | 0.832 | 4.638(4.229 ,5.085) | <0.001*** | 0.843 | 3.063(2.698 ,3.477) | <0.001*** | 0.912 | 3.075(2.688 ,3.517) | <0.001*** | 0.911 |
|  | VAI | 3.971(3.646 ,4.324) | <0.001*** | 0.817 | 4.246(3.887 ,4.637) | <0.001*** | 0.833 | 2.821(2.431 ,3.273) | <0.001*** | 0.903 | 2.774(2.370 ,3.245) | <0.001*** | 0.901 |
|  | CVAI | 3.565(3.294 ,3.859) | <0.001*** | 0.802 | 4.116(3.779 ,4.482) | <0.001*** | 0.822 | 2.439(2.198 ,2.706) | <0.001*** | 0.911 | 2.506(2.243 ,2.800) | <0.001*** | 0.910 |
|  | TyG | 4.284(3.924 ,4.677) | <0.001*** | 0.830 | 4.341(3.972 ,4.744) | <0.001*** | 0.838 | 3.098(2.685 ,3.576) | <0.001*** | 0.905 | 3.113(2.677 ,3.620) | <0.001*** | 0.904 |
|  | BMI | 2.535(2.362 ,2.721) | <0.001*** | 0.741 | 2.601(2.419 ,2.795) | <0.001*** | 0.752 | 1.946(1.779 ,2.129) | <0.001*** | 0.906 | 1.981(1.802 ,2.178) | <0.001*** | 0.906 |
|  | WC | 2.682(2.500 ,2.877) | <0.001*** | 0.758 | 2.715(2.529 ,2.916) | <0.001*** | 0.766 | 2.075(1.902 ,2.265) | <0.001*** | 0.911 | 2.078(1.895 ,2.278) | <0.001*** | 0.909 |
| China-2020 | LAP | 5.191(4.720 ,5.709) | <0.001*** | 0.849 | 5.540(5.022 ,6.111) | <0.001*** | 0.867 | 3.961(3.452 ,4.547) | <0.001*** | 0.929 | 4.404(3.771 ,5.144) | <0.001*** | 0.934 |
|  | VAI | 4.276(3.921 ,4.662) | <0.001*** | 0.828 | 4.934(4.496 ,5.416) | <0.001*** | 0.851 | 3.380(2.889 ,3.956) | <0.001*** | 0.917 | 3.738(3.143 ,4.445) | <0.001*** | 0.922 |
|  | CVAI | 3.668(3.390 ,3.969) | <0.001*** | 0.808 | 4.815(4.399 ,5.271) | <0.001*** | 0.846 | 2.864(2.568 ,3.195) | <0.001*** | 0.926 | 2.983(2.648 ,3.361) | <0.001*** | 0.930 |
|  | TyG | 5.076(4.623 ,5.572) | <0.001*** | 0.850 | 5.072(4.618 ,5.569) | <0.001*** | 0.856 | 4.033(3.453 ,4.711) | <0.001*** | 0.921 | 4.320(3.646 ,5.117) | <0.001*** | 0.925 |
|  | BMI | 2.674(2.491 ,2.871) | <0.001*** | 0.750 | 2.784(2.588 ,2.995) | <0.001*** | 0.767 | 2.179(1.983 ,2.394) | <0.001*** | 0.920 | 2.198(1.985 ,2.433) | <0.001*** | 0.924 |
|  | WC | 3.046(2.832 ,3.277) | <0.001*** | 0.781 | 3.046(2.831 ,3.278) | <0.001*** | 0.787 | 2.504(2.279 ,2.750) | <0.001*** | 0.927 | 2.511(2.268 ,2.781) | <0.001*** | 0.930 |
| *Notes: BMI, body mass index; CVAI, Chinese visceral adiposity index; LAP, lipid accumulation product; MetS, metabolic syndrome; TyG, triglyceride glucose; VAI, visceral adiposity index; WC, waist circumference; AUC, area under the curve;***:p<0.001(extreme significant) Model 1: Unadjusted model  Model 2: Model 1+ age, gender, marital status, education level, smoking, and drinking  Model 3: Model 2 + SBP, DBP, TG, HDL-C, and FBG Model 4: Model 3 + place of residence, social activities, and health insurance* | | | | | | | | | | | | |  |

| **Table S4**　Logistic regression analysis of LAP, VAI, CVAI, and TyG parameters predicting the risk of MetS (in age>=60) | | | | | | | | | | | | |  |
| --- | --- | --- | --- | --- | --- | --- | --- | --- | --- | --- | --- | --- | --- |
| criterion | index | model1 | | | model2 | | | model3 | | | model4 | | |
|  |  | OR(95%CI) | *P-value* | AUC | OR(95%CI) | *P-value* | AUC | OR(95%CI) | *P-value* | AUC | OR(95%CI) | *P-value* | AUC |
| IDF | LAP | 6.628(6.032 ,7.283 ) | <0.001*** | 0.889 | 6.248(5.673 ,6.881 ) | <0.001*** | 0.904 | 7.717(6.700 ,8.889 ) | <0.001*** | 0.927 | 7.639(6.606 ,8.833 ) | <0.001*** | 0.928 |
|  | VAI | 4.147(3.850 ,4.466 ) | <0.001*** | 0.840 | 3.817(3.535 ,4.121 ) | <0.001*** | 0.855 | 3.172(2.775 ,3.627 ) | <0.001*** | 0.881 | 3.133(2.728 ,3.599 ) | <0.001*** | 0.885 |
|  | CVAI | 8.102(7.290 ,9.005 ) | <0.001*** | 0.904 | 10.512(9.275 ,11.913 ) | <0.001*** | 0.920 | 7.991(6.976 ,9.154 ) | <0.001*** | 0.937 | 7.739(6.740 ,8.886 ) | <0.001*** | 0.938 |
|  | TyG | 3.170(2.968 ,3.386 ) | <0.001*** | 0.796 | 3.131(2.920 ,3.356 ) | <0.001*** | 0.836 | 2.411(2.138 ,2.718 ) | <0.001*** | 0.873 | 2.368(2.092 ,2.681 ) | <0.001*** | 0.878 |
|  | BMI | 3.452(3.225 ,3.694 ) | <0.001*** | 0.814 | 3.691(3.424 ,3.979 ) | <0.001*** | 0.859 | 3.239(2.971 ,3.532 ) | <0.001*** | 0.913 | 3.215(2.942 ,3.514 ) | <0.001*** | 0.915 |
|  | WC | 4.497(4.163 ,4.858 ) | <0.001*** | 0.852 | 5.456(4.984 ,5.973 ) | <0.001*** | 0.900 | 5.151(4.638 ,5.721 ) | <0.001*** | 0.938 | 5.048(4.536 ,5.619 ) | <0.001*** | 0.939 |
| NCEP ATPIII | LAP | 4.951(4.540 ,5.399 ) | <0.001*** | 0.857 | 5.144(4.697 ,5.633 ) | <0.001*** | 0.866 | 3.348(2.957 ,3.792 ) | <0.001*** | 0.916 | 3.369(2.965 ,3.828 ) | <0.001*** | 0.916 |
|  | VAI | 4.256(3.928 ,4.611 ) | <0.001*** | 0.841 | 4.572(4.192 ,4.988 ) | <0.001*** | 0.850 | 2.662(2.283 ,3.103 ) | <0.001*** | 0.901 | 2.607(2.229 ,3.048 ) | <0.001*** | 0.901 |
|  | CVAI | 3.645(3.375 ,3.936 ) | <0.001*** | 0.818 | 4.613(4.207 ,5.058 ) | <0.001*** | 0.835 | 2.771(2.483 ,3.093 ) | <0.001*** | 0.916 | 2.808(2.508 ,3.145 ) | <0.001*** | 0.916 |
|  | TyG | 4.406(4.058 ,4.784 ) | <0.001*** | 0.842 | 4.375(4.022 ,4.759 ) | <0.001*** | 0.856 | 2.674(2.309 ,3.095 ) | <0.001*** | 0.899 | 2.687(2.315 ,3.119 ) | <0.001*** | 0.900 |
|  | BMI | 2.657(2.492 ,2.833 ) | <0.001*** | 0.766 | 2.695(2.521 ,2.882 ) | <0.001*** | 0.786 | 2.230(2.048 ,2.429 ) | <0.001*** | 0.916 | 2.266(2.076 ,2.474 ) | <0.001*** | 0.916 |
|  | WC | 2.833(2.651 ,3.027 ) | <0.001*** | 0.777 | 2.848(2.660 ,3.049 ) | <0.001*** | 0.798 | 2.363(2.169 ,2.574 ) | <0.001*** | 0.920 | 2.381(2.180 ,2.599 ) | <0.001*** | 0.919 |
| China-2020 | LAP | 5.710(5.197 ,6.274 ) | <0.001*** | 0.872 | 6.648(5.996 ,7.370 ) | <0.001*** | 0.887 | 4.764(4.128 ,5.497 ) | <0.001*** | 0.935 | 4.718(4.074 ,5.464 ) | <0.001*** | 0.934 |
|  | VAI | 4.570(4.201 ,4.971 ) | <0.001*** | 0.849 | 5.668(5.145 ,6.244 ) | <0.001*** | 0.869 | 3.511(2.976 ,4.141 ) | <0.001*** | 0.918 | 3.515(2.970 ,4.161 ) | <0.001*** | 0.917 |
|  | CVAI | 3.669(3.393 ,3.969 ) | <0.001*** | 0.816 | 5.797(5.227 ,6.428 ) | <0.001*** | 0.846 | 3.669(3.240 ,4.155 ) | <0.001*** | 0.935 | 3.665(3.226 ,4.164 ) | <0.001*** | 0.934 |
|  | TyG | 5.254(4.798 ,5.754 ) | <0.001*** | 0.862 | 5.364(4.886 ,5.888 ) | <0.001*** | 0.869 | 3.522(3.002 ,4.132 ) | <0.001*** | 0.916 | 3.563(3.030 ,4.191 ) | <0.001*** | 0.916 |
|  | BMI | 2.744(2.570 ,2.930 ) | <0.001*** | 0.773 | 2.802(2.618 ,3.000 ) | <0.001*** | 0.785 | 2.433(2.220 ,2.666 ) | <0.001*** | 0.930 | 2.442(2.223 ,2.682 ) | <0.001*** | 0.930 |
|  | WC | 3.189(2.971 ,3.423 ) | <0.001*** | 0.798 | 3.178(2.958 ,3.415 ) | <0.001*** | 0.809 | 2.903(2.637 ,3.197 ) | <0.001*** | 0.938 | 2.892(2.621 ,3.192 ) | <0.001*** | 0.937 |
| *Notes: BMI, body mass index; CVAI, Chinese visceral adiposity index; LAP, lipid accumulation product; MetS, metabolic syndrome; TyG, triglyceride glucose; VAI, visceral adiposity index; WC, waist circumference; AUC, area under the curve;***:p<0.001(extreme significant) Model 1: Unadjusted model  Model 2: Model 1+ age, gender, marital status, education level, smoking, and drinking  Model 3: Model 2 + SBP, DBP, TG, HDL-C, and FBG Model 4: Model 3 + place of residence, social activities, and health insurance* | | | | | | | | | | | | |  |

| Table S5 ROC analysis of six body fat metrics to predict the risk of MetS (in male) | | | | | | | | | |
| --- | --- | --- | --- | --- | --- | --- | --- | --- | --- |
| criterion | index | AUC(95%CI) | Sensitivity(%) | Specificity(%) | LR+ | LR- | Threshold | Youden index | P-value |
| IDF | LAP | 0.921 (0.914,0.929) | 84.90% | 83.27% | 5.076 | 0.181 | 35.990 | 0.682 | <0.001*** |
|  | VAI | 0.845 (0.834,0.857) | 78.88% | 75.86% | 3.268 | 0.278 | 3.750 | 0.547 | <0.001*** |
|  | **CVAI** | **0.951 (0.945,0.956)** | 93.92% | 85.39% | 6.426 | 0.071 | 138.700 | 0.793 | <0.001*** |
|  | TyG | 0.823 (0.811,0.835) | 77.67% | 75.59% | 3.182 | 0.296 | 8.780 | 0.533 | <0.001*** |
|  | BMI | 0.895 (0.886,0.904) | 87.40% | 78.78% | 4.119 | 0.160 | 24.230 | 0.662 | <0.001*** |
|  | **WC** | **0.948 (0.942,0.954)** | 100.00% | 86.98% | 7.682 | 0.000 | 90.000 | 0.870 | <0.001*** |
| NCEP ATPIII | LAP | 0.861 (0.848,0.874) | 80.13% | 79.71% | 3.950 | 0.249 | 36.780 | 0.598 | <0.001*** |
|  | **VAI** | **0.873 (0.861,0.884)** | 81.62% | 79.66% | 4.013 | 0.231 | 3.900 | 0.613 | <0.001*** |
|  | CVAI | 0.833 (0.820,0.846) | 73.37% | 78.42% | 3.400 | 0.340 | 142.610 | 0.518 | <0.001*** |
|  | **TyG** | **0.870 (0.857,0.881)** | 78.65% | 83.89% | 4.881 | 0.255 | 8.940 | 0.625 | <0.001*** |
|  | BMI | 0.773 (0.758,0.788) | 72.30% | 69.17% | 2.345 | 0.401 | 24.110 | 0.415 | <0.001*** |
|  | WC | 0.785 (0.770,0.800) | 75.02% | 68.70% | 2.397 | 0.364 | 88.400 | 0.437 | <0.001*** |
| China-2020 | **LAP** | **0.898 (0.889,0.908)** | 84.67% | 80.81% | 4.413 | 0.190 | 34.460 | 0.655 | <0.001*** |
|  | **VAI** | **0.890 (0.880,0.900)** | 82.49% | 80.54% | 4.240 | 0.218 | 3.780 | 0.630 | <0.001*** |
|  | CVAI | 0.876 (0.866,0.887) | 85.47% | 77.69% | 3.831 | 0.187 | 134.700 | 0.632 | <0.001*** |
|  | TyG | 0.882 (0.872,0.892) | 85.68% | 78.39% | 3.965 | 0.183 | 8.780 | 0.641 | <0.001*** |
|  | BMI | 0.812 (0.799,0.824) | 74.49% | 75.67% | 3.062 | 0.337 | 24.390 | 0.502 | <0.001*** |
|  | WC | 0.841 (0.829,0.853) | 81.10% | 79.49% | 3.955 | 0.238 | 90.000 | 0.606 | <0.001*** |
| Notes: AUC, area under the curve; BMI, body mass index; LAP, lipid accumulation product; CVAI, Chinese visceral adiposity index; MetS, metabolic syndrome; TyG, triglyceride glucose; VAI, visceral adiposity index; LR+ :Positive likelihood ratio; LR-: Negative likelihood ratio | | | | | | | | | |

| **Table S6** ROC analysis of six body fat metrics to predict the risk of MetS (in female) | | | | | | | | | |
| --- | --- | --- | --- | --- | --- | --- | --- | --- | --- |
| criterion | index | AUC(95%CI) | Sensitivity(%) | Specificity(%) | LR+ | LR- | Threshold | Youden index | P-value |
| IDF | **LAP** | **0.903 (0.898 ,0.909)** | 81.39% | 81.67% | 4.440 | 0.228 | 37.630 | 0.631 | <0.001*** |
|  | VAI | 0.854 (0.846 ,0.861) | 78.18% | 77.41% | 3.461 | 0.282 | 4.200 | 0.556 | <0.001*** |
|  | **CVAI** | **0.909 (0.904 ,0.914)** | 90.36% | 76.53% | 3.850 | 0.126 | 147.600 | 0.669 | <0.001*** |
|  | TyG | 0.815 (0.807 ,0.823) | 73.46% | 77.75% | 3.302 | 0.341 | 8.780 | 0.512 | <0.001*** |
|  | BMI | 0.822 (0.814 ,0.830) | 77.18% | 73.00% | 2.858 | 0.313 | 24.000 | 0.502 | <0.001*** |
|  | WC | 0.860 (0.853 ,0.867) | 72.65% | 84.71% | 4.752 | 0.323 | 89.400 | 0.574 | <0.001*** |
| NCEP ATPIII | **LAP** | **0.862 (0.854 ,0.870)** | 80.84% | 77.76% | 3.635 | 0.246 | 42.010 | 0.586 | <0.001*** |
|  | VAI | 0.846 (0.838 ,0.854) | 79.77% | 75.29% | 3.229 | 0.269 | 4.590 | 0.551 | <0.001*** |
|  | CVAI | 0.832 (0.823 ,0.840) | 75.00% | 76.67% | 3.215 | 0.326 | 162.300 | 0.517 | <0.001*** |
|  | **TyG** | **0.856 (0.848 ,0.863)** | 84.54% | 79.44% | 4.113 | 0.195 | 8.850 | 0.640 | <0.001*** |
|  | BMI | 0.765 (0.755 ,0.775) | 70.03% | 70.89% | 2.406 | 0.423 | 24.650 | 0.409 | <0.001*** |
|  | WC | 0.786 (0.776 ,0.795) | 78.83% | 68.17% | 2.477 | 0.311 | 87.700 | 0.470 | <0.001*** |
| China-2020 | **LAP** | **0.881 (0.874 ,0.888)** | 80.14% | 81.65% | 4.369 | 0.243 | 44.760 | 0.618 | <0.001*** |
|  | VAI | 0.860 (0.852 ,0.867) | 80.82% | 76.81% | 3.485 | 0.250 | 4.660 | 0.576 | <0.001*** |
|  | CVAI | 0.831 (0.823 ,0.839) | 84.09% | 66.80% | 2.533 | 0.238 | 150.900 | 0.509 | <0.001*** |
|  | **TyG** | **0.879 (0.872 ,0.886)** | 84.54% | 79.44% | 4.113 | 0.195 | 8.850 | 0.640 | <0.001*** |
|  | BMI | 0.775 (0.766 ,0.784) | 77.26% | 66.13% | 2.281 | 0.344 | 23.420 | 0.434 | <0.001*** |
|  | WC | 0.808 (0.799 ,0.816) | 79.33% | 70.92% | 2.729 | 0.291 | 88.100 | 0.503 | <0.001*** |
| Notes: AUC, area under the curve; BMI, body mass index; LAP, lipid accumulation product; CVAI, Chinese visceral adiposity index; MetS, metabolic syndrome; TyG, triglyceride glucose; VAI, visceral adiposity index; LR+ :Positive likelihood ratio; LR-: Negative likelihood ratio | | | | | | | | | |

| **Table S7** ROC analysis of six body fat metrics to predict the risk of MetS (in age<60) | | | | | | | | | |
| --- | --- | --- | --- | --- | --- | --- | --- | --- | --- |
| criterion | index | AUC(95%CI) | Sensitivity(%) | Specificity(%) | LR+ | LR- | Threshold | Youden index | P-value |
| IDF | **LAP** | **0.896 (0.888,0.904)** | 78.79% | 83.73% | 4.841 | 0.253 | 41.530 | 0.625 | <0.001*** |
|  | VAI | 0.848 (0.838,0.859) | 79.80% | 75.00% | 3.192 | 0.269 | 5.100 | 0.548 | <0.001*** |
|  | **CVAI** | **0.906 (0.899,0.914)** | 87.79% | 78.95% | 4.170 | 0.155 | 145.600 | 0.667 | <0.001*** |
|  | TyG | 0.815 (0.803,0.826) | 78.09% | 73.15% | 2.909 | 0.300 | 8.720 | 0.512 | <0.001*** |
|  | BMI | 0.815 (0.804,0.826) | 79.62% | 70.73% | 2.721 | 0.288 | 24.250 | 0.504 | <0.001*** |
|  | WC | 0.848 (0.838,0.858) | 81.60% | 71.85% | 2.898 | 0.256 | 86.900 | 0.535 | <0.001*** |
| NCEP ATPIII | **LAP** | **0.855 (0.844,0.867)** | 78.71% | 79.07% | 3.761 | 0.269 | 47.650 | 0.578 | <0.001*** |
|  | VAI | 0.837 (0.825,0.849) | 82.12% | 70.45% | 2.779 | 0.254 | 4.570 | 0.526 | <0.001*** |
|  | CVAI | 0.821 (0.808,0.834) | 73.56% | 76.96% | 3.193 | 0.344 | 158.400 | 0.505 | <0.001*** |
|  | **TyG** | **0.852 (0.841,0.863)** | 83.44% | 74.37% | 3.256 | 0.223 | 8.850 | 0.578 | <0.001*** |
|  | BMI | 0.764 (0.750,0.777) | 76.62% | 64.95% | 2.186 | 0.360 | 24.670 | 0.416 | <0.001*** |
|  | WC | 0.779 (0.765,0.793) | 81.07% | 64.47% | 2.282 | 0.294 | 87.600 | 0.455 | <0.001*** |
| China-2020 | **LAP** | **0.876 (0.866,0.886)** | 83.86% | 77.29% | 3.692 | 0.209 | 43.900 | 0.612 | <0.001*** |
|  | VAI | 0.852 (0.841,0.862) | 82.74% | 72.96% | 3.060 | 0.237 | 4.660 | 0.557 | <0.001*** |
|  | CVAI | 0.827 (0.815,0.838) | 81.42% | 70.65% | 2.774 | 0.263 | 150.900 | 0.521 | <0.001*** |
|  | **TyG** | **0.874 (0.864,0.883)** | 86.64% | 76.59% | 3.702 | 0.174 | 8.850 | 0.632 | <0.001*** |
|  | BMI | 0.770 (0.757,0.783) | 77.58% | 66.22% | 2.297 | 0.339 | 24.680 | 0.438 | <0.001*** |
|  | WC | 0.800 (0.788,0.813) | 82.41% | 65.86% | 2.414 | 0.267 | 87.600 | 0.483 | <0.001*** |
| Notes: AUC, area under the curve; BMI, body mass index; LAP, lipid accumulation product; CVAI, Chinese visceral adiposity index; MetS, metabolic syndrome; TyG, triglyceride glucose; VAI, visceral adiposity index; LR+ :Positive likelihood ratio; LR-: Negative likelihood ratio | | | | | | | | | |

| **Table S8** ROC analysis of six body fat metrics to predict the risk of MetS (in age>=60) | | | | | | | | | |
| --- | --- | --- | --- | --- | --- | --- | --- | --- | --- |
| criterion | index | AUC(95%CI) | Sensitivity(%) | Specificity(%) | LR+ | LR- | Threshold | Youden index | P-value |
| IDF | **LAP** | **0.912 (0.904 ,0.919)** | 82.11% | 82.23% | 4.620 | 0.218 | 35.920 | 0.643 | <0.001*** |
|  | VAI | 0.859 (0.849 ,0.869) | 79.08% | 77.53% | 3.519 | 0.270 | 3.980 | 0.566 | <0.001*** |
|  | **CVAI** | **0.923 (0.916 ,0.930)** | 87.84% | 81.50% | 4.748 | 0.149 | 159.400 | 0.693 | <0.001*** |
|  | TyG | 0.814 (0.802 ,0.825) | 72.21% | 78.83% | 3.411 | 0.353 | 8.760 | 0.510 | <0.001*** |
|  | BMI | 0.831 (0.820 ,0.841) | 76.76% | 74.40% | 2.999 | 0.312 | 23.600 | 0.512 | <0.001*** |
|  | WC | 0.871 (0.862 ,0.880) | 72.65% | 84.71% | 4.752 | 0.323 | 89.400 | 0.574 | <0.001*** |
| NCEP ATPIII | **LAP** | **0.876 (0.865 ,0.886)** | 82.96% | 78.23% | 3.811 | 0.218 | 38.140 | 0.612 | <0.001*** |
|  | VAI | 0.861 (0.851 ,0.872) | 77.91% | 80.36% | 3.966 | 0.275 | 4.600 | 0.583 | <0.001*** |
|  | CVAI | 0.843 (0.831 ,0.854) | 87.84% | 81.50% | 4.748 | 0.149 | 159.400 | 0.693 | <0.001*** |
|  | **TyG** | **0.864 (0.853 ,0.875)** | 78.62% | 81.74% | 4.305 | 0.262 | 8.850 | 0.604 | <0.001*** |
|  | BMI | 0.780 (0.767 ,0.793) | 74.98% | 67.45% | 2.304 | 0.371 | 23.620 | 0.424 | <0.001*** |
|  | WC | 0.795 (0.782 ,0.808) | 72.65% | 84.71% | 4.752 | 0.323 | 89.400 | 0.574 | <0.001*** |
| China-2020 | **LAP** | **0.890 (0.881 ,0.900)** | 84.68% | 78.51% | 3.941 | 0.195 | 38.450 | 0.632 | <0.001*** |
|  | VAI | 0.871 (0.861 ,0.881) | 78.99% | 80.76% | 4.105 | 0.260 | 4.660 | 0.598 | <0.001*** |
|  | CVAI | 0.840 (0.829 ,0.851) | 72.66% | 78.90% | 3.444 | 0.347 | 171.400 | 0.516 | <0.001*** |
|  | **TyG** | **0.885 (0.876 ,0.895)** | 82.84% | 82.04% | 4.611 | 0.209 | 8.840 | 0.649 | <0.001*** |
|  | BMI | 0.788 (0.775 ,0.800) | 77.28% | 67.60% | 2.385 | 0.336 | 23.600 | 0.449 | <0.001*** |
|  | WC | 0.816 (0.804 ,0.828) | 77.34% | 75.38% | 3.141 | 0.301 | 88.700 | 0.527 | <0.001*** |
| Notes: AUC, area under the curve; BMI, body mass index; LAP, lipid accumulation product; CVAI, Chinese visceral adiposity index; MetS, metabolic syndrome; TyG, triglyceride glucose; VAI, visceral adiposity index; LR+ :Positive likelihood ratio; LR-: Negative likelihood ratio | | | | | | | | | |

| **Table S9.** Pairwise comparisons of the AUCs of six body fat metrics in predicting Mets by the Delong test.(in male) | | | | | | |
| --- | --- | --- | --- | --- | --- | --- |
| criterion | Variable | LAP | VAI | CVAI | TyG | BMI |
| IDF | VAI | 22.93*** |  |  |  |  |
|  | CVAI | 7.907*** | 18.139*** |  |  |  |
|  | TyG | 24.496*** | 5.997*** | 19.359*** |  |  |
|  | BMI | 5.185*** | 6.874*** | 14.519*** | 9.43*** |  |
|  | WC | 6.508*** | 15.993*** | 1.506 | 17.82*** | 14.683*** |
| NCEP ATPIII | VAI | 2.571*** |  |  |  |  |
|  | CVAI | 6.225*** | 5.677*** |  |  |  |
|  | TyG | 1.454 | 0.928 | 4.385*** |  |  |
|  | BMI | 12.668*** | 11.466*** | 11.047*** | 10.496*** |  |
|  | WC | 13.926*** | 10.561*** | 19.896*** | 9.109*** | 2.519* |
| China-2020 | VAI | 2.228* |  |  |  |  |
|  | CVAI | 5.392*** | 2.115* |  |  |  |
|  | TyG | 3.33** | 2.063* | 0.812 |  |  |
|  | BMI | 13.785*** | 9.97*** | 12.977*** | 8.675*** |  |
|  | WC | 11.799*** | 6.585*** | 16.058*** | 5.104*** | 6.378*** |
| Notes: AUC, area under the curve; BMI, body mass index; LAP, lipid accumulation product; CVAI, Chinese visceral adiposity index; MetS, metabolic syndrome; TyG, triglyceride glucose; VAI, visceral adiposity index; ***P< 0.001, **P< 0.01,*P< 0.05 | | | | | | |

| **Table S10.** Pairwise comparisons of the AUCs of six body fat metrics in predicting Mets by the Delong test(in female) | | | | | | |
| --- | --- | --- | --- | --- | --- | --- |
| criterion | Variable | LAP | VAI | CVAI | TyG | BMI |
| IDF | VAI | 22.93^***^ |  |  |  |  |
|  | CVAI | 7.907^***^ | 18.139^***^ |  |  |  |
|  | TyG | 24.496^***^ | 5.997^***^ | 19.359^***^ |  |  |
|  | BMI | 5.185^***^ | 6.874^***^ | 14.519^***^ | 9.43^***^ |  |
|  | WC | 6.508^***^ | 15.993^***^ | 1.506 | 17.82^***^ | 14.683^***^ |
| NCEP ATPIII | VAI | 2.571^***^ |  |  |  |  |
|  | CVAI | 6.225^***^ | 5.677^***^ |  |  |  |
|  | TyG | 1.454 | 0.928 | 4.385^***^ |  |  |
|  | BMI | 12.668^***^ | 11.466^***^ | 11.047^***^ | 10.496^***^ |  |
|  | WC | 13.926^***^ | 10.561^***^ | 19.896^***^ | 9.109^***^ | 2.519^*^ |
| China-2020 | VAI | 2.228^*^ |  |  |  |  |
|  | CVAI | 5.392^***^ | 2.115^*^ |  |  |  |
|  | TyG | 3.33^**^ | 2.063^*^ | 0.812 |  |  |
|  | BMI | 13.785^***^ | 9.97^***^ | 12.977^***^ | 8.675^***^ |  |
|  | WC | 11.799^***^ | 6.585^***^ | 16.058^***^ | 5.104^***^ | 6.378^***^ |
| Notes: AUC, area under the curve; BMI, body mass index; LAP, lipid accumulation product; CVAI, Chinese visceral adiposity index; MetS, metabolic syndrome; TyG, triglyceride glucose; VAI, visceral adiposity index; ***P< 0.001, **P< 0.01,*P< 0.05 | | | | | | |

| **Table S11.** Pairwise comparisons of the AUCs of six body fat metrics in predicting Mets by the Delong test.(in age <60) | | | | | | |
| --- | --- | --- | --- | --- | --- | --- |
| criterion | Variable | LAP | VAI | CVAI | TyG | BMI |
| IDF | VAI | 15.183^***^ |  |  |  |  |
|  | CVAI | 2.771^*^ | 10.797^***^ |  |  |  |
|  | TyG | 20.855^***^ | 9.07^***^ | 14.162^***^ |  |  |
|  | BMI | 14.112^***^ | 4.412^***^ | 21.924^***^ | 0.0574 |  |
|  | WC | 9.74^***^ | 0.0344 | 15.893^***^ | 4.47^***^ | 8.074^***^ |
| NCEP ATPIII | VAI | 4.607^***^ |  |  |  |  |
|  | CVAI | 7.068^***^ | 2.382^*^ |  |  |  |
|  | TyG | 0.667 | 3.833^***^ | 3.958^***^ |  |  |
|  | BMI | 12.976^***^ | 8.36^***^ | 11.199^***^ | 9.985^***^ |  |
|  | WC | 12.979^***^ | 6.919^***^ | 9.1^***^ | 8.3^***^ | 3.029^*^ |
| China-2020 | VAI | 6.864^***^ |  |  |  |  |
|  | CVAI | 10.668^***^ | 4.06^***^ |  |  |  |
|  | TyG | 0.457 | 5.701^***^ | 6.73^***^ |  |  |
|  | BMI | 15.771^***^ | 9.836^***^ | 11.48^***^ | 12.861^***^ |  |
|  | WC | 13.379^***^ | 6.512^***^ | 5.961^***^ | 9.267^***^ | 6.12^***^ |
| Notes: AUC, area under the curve; BMI, body mass index; LAP, lipid accumulation product; CVAI, Chinese visceral adiposity index; MetS, metabolic syndrome; TyG, triglyceride glucose; VAI, visceral adiposity index; ***P< 0.001, **P< 0.01,*P< 0.05 | | | | | | |

| **Table S12.** Pairwise comparisons of the AUCs of six body fat metrics in predicting Mets by the Delong test.(in age >=60) | | | | | | |
| --- | --- | --- | --- | --- | --- | --- |
| criterion | Variable | LAP | VAI | CVAI | TyG | BMI |
| IDF | VAI | 16.85^***^ |  |  |  |  |
|  | CVAI | 3.175^*^ | 12.877^***^ |  |  |  |
|  | TyG | 23.904^***^ | 11.654^***^ | 17.303^***^ |  |  |
|  | BMI | 14.914^***^ | 3.843^**^ | 19.481^***^ | 2.188^*^ |  |
|  | WC | 9.05^***^ | 1.829 | 12.715^***^ | 7.777^***^ | 10.092^***^ |
| NCEP ATPIII | VAI | 3.853^**^ |  |  |  |  |
|  | CVAI | 7.479^***^ | 3.137^*^ |  |  |  |
|  | TyG | 2.417^*^ | 0.677 | 2.931^*^ |  |  |
|  | BMI | 14.576^***^ | 9.967^***^ | 11.252^***^ | 9.898^***^ |  |
|  | WC | 14.956^***^ | 8.637^***^ | 9.791^***^ | 8.221^***^ | 3.241^*^ |
| China-2020 | VAI | 5.339^***^ |  |  |  |  |
|  | CVAI | 11.328^***^ | 5.361^***^ |  |  |  |
|  | TyG | 1.133 | 3.741^*^ | 6.608^***^ |  |  |
|  | BMI | 15.771^***^ | 10.343^***^ | 9.365^***^ | 12.06^***^ |  |
|  | WC | 13.887^***^ | 7.264^***^ | 4.827^***^ | 8.696^***^ | 6.084^***^ |
| Notes: AUC, area under the curve; BMI, body mass index; LAP, lipid accumulation product; CVAI, Chinese visceral adiposity index; MetS, metabolic syndrome; TyG, triglyceride glucose; VAI, visceral adiposity index; ***P< 0.001, **P< 0.01,*P< 0.05 | | | | | | |
